# Diagnostic accuracy of physical examination tests for painful cervical radiculopathy: update of a systematic review and meta-analysis

**DOI:** 10.1186/s12891-026-09551-0

**Published:** 2026-02-13

**Authors:** Erik J Thoomes, Michail Arvanitidis, Sarita van Geest, Danielle A van der Windt, Arianne P Verhagen, Marloes de Graaf, Barbara Kuijper, Gwendolyne G M Scholten-Peeters, Carmen L Vleggeert-Lankamp, Deborah Falla

**Affiliations:** 1 Centre of Precision Rehabilitation for Spinal Pain (CPR Spine), School of Sport, Exercise and Rehabilitation Sciences, College of Life and Environmental Sciences, University of Birmingham, Birmingham, UK; 2 De Singel Physiotherapy clinic, Leiden, The Netherlands; 3 Department of Neurology, Martini Ziekenhuis, Groningen, The Netherlands; 4 Keele University, School of Medicine, Staffordshire, UK; 5 University of Technology Sydney, Graduate School of Health, Sydney, Australia; 6 Department of Manual Therapy, Breederode University of Applied Science, Rotterdam, The Netherlands; 7 Department of Neurology, Maasstad Hospital, Rotterdam, The Netherlands; 8 Department of Human Movement Sciences, Faculty of Behavioural and Movement Sciences, Program Musculoskeletal Health, Vrije Universiteit Amsterdam, Amsterdam, The Netherlands; 9 Department of Neurosurgery, Leiden University Medical Centre, Leiden, The Netherlands

**Keywords:** Cervical radiculopathy, Diagnostic accuracy, Physical examination tests, Meta-analysis, Systematic review

## Abstract

**Background:**

Cervical radiculopathy (CR) is a clinical condition caused by compression of the nerve root. In clinical practice, the diagnosis of CR is based on information from the patient’s history, physical examination, and diagnostic imaging. This systematic review aimed to update and summarise the evidence reported in a systematic review published in 2018 on the diagnostic performance of physical examination tests.

**Methods:**

A literature search was performed in six electronic databases. Selection, assessment of risk of bias (using the QUADAS-2) and data extraction were performed independently by two reviewers. Sensitivity and specificity were calculated, and the certainty of the evidence was assessed using the GRADE framework. For the meta-analysis, a hierarchical bivariate random-effects model was used and, in line with recommendations for sparse data, models were interpreted as bivariate fixed-effect Generalized Linear Mixed Models.

**Results:**

In total, eight studies were included. Diagnostic value was assessed for six physical examination tests. Slightly different versions of Spurling’s test were assessed in five studies, with a reported high specificity ranging from 0.84 to 1.00 (95% CI range: 0.56–1.00) and sensitivity values ranging from 0.38 to 0.98 (95%CI range: 0.22–0.99). There is low certainty evidence of pooled sensitivity of 0.70 (95%CI 0.60–0.79) and specificity of 0.71 (95%CI 0.63–0.79) for Upper Limb Neurodynamic Test (ULNT) 1. Similary there was low certainty evidence of pooled sensitivity of 0.97 (95%CI 0.88–0.99) and pooled specificity of 0.51 (95%CI 0.40–0.62) for combined ULNTs, and of pooled sensitivity of 0.49 (95%CI 0.39–0.60) and pooled specificity of 0.76 (95%CI 0.66–0.84) for the shoulder abduction relief test. All other tests were assessed in a single study only.

**Conclusions:**

Clinicians may use the outcome of Spurling’s test and the outcome of the combination of four ULNTs to assist their clinical reasoning in diagnosing CR. However, evidence on the diagnostic accuracy of all physical tests for the diagnosis of CR is sparse, and the certainty of the evidence was very low for all outcomes of all tests, implying that new studies of high methodological value are still required to strengthen these results. Because of the small number of studies, the pooled estimates are valid only for the populations and tests studied in the specific studies included in this review.

**Supplementary Information:**

The online version contains supplementary material available at 10.1186/s12891-026-09551-0.

## Introduction

Cervical radiculopathy (CR) is a clinical condition characterised by motor, reflex and/or sensory changes such as paraesthesia or numbness, often provoked by neck posture(s) and/or movement(s) [1]. CR is usually caused by compression of the nerve root due to cervical disc herniation or degenerative spondylotic changes, but radicular symptoms can also occur without evident compression, for instance, due to inflammation of the nerve [2].

Clinically, radiculopathy needs to be differentiated from radicular pain. Radicular pain is pain evoked by ectopic discharges emanating from a dorsal root or its ganglion. The evoked painful sensation is often described as “lancinating”, “shocking”, or “electric” [2–4]. In contrast, radiculopathy is a neurological state in which conduction is blocked along a spinal nerve or its roots, resulting in numbness as a symptom or, when motor fibres are blocked, weakness ensues. Diminished reflexes occur as a result of a sensory or motor block [2–4]. Additionally, mixed pain presentations with nociceptive and/or neuropathic components are common. For instance, radicular pain can occur with radiculopathy and/or with heightened nerve mechanosensitivity [5].

Epidemiological data on CR are sparse, and a recent systematic review, aiming to determine the incidence and/or prevalence of CR in adults, stated that contemporary population-based data are lacking [6]. Most research still relies on one population-based study in Rochester, Minnesota (USA), conducted in 1996. That study reported that CR has an annual incidence rate of 107.3 per 100,000 for men and 63.5 per 100,000 for women [1].

Most patients with neck and radiating arm pain typically first consult a primary health care practitioner (e.g., a General Practitioner, Physiotherapist, Osteopath or Chiropractor) [7–9]. Following history taking, clinicians formulate initial hypotheses concerning the cause of the patient’s problem, and subsequently plan a physical examination to substantiate one (or more) of the initially formed hypotheses [9–12]. In patients presenting with radiating neck and arm pain, it is important to differentiate between somatic referred pain and radicular pain caused by an encroached cervical nerve root, as both the prognosis and the management strategies will differ [13, 14]. In patients with suspected CR, several physical tests have been proposed, aiming to provoke or decrease patient-specific signs and symptoms, thereby increasing the likelihood of CR being the cause of the complaints [15–17]. Although there is no diagnostic “gold standard”, a combination of history taking, physical examination, including neurological assessment and Magnetic Resonance Imaging (MRI) is generally considered best evidence-based clinical practice in primary and secondary health care [18–20].

In 2018, a systematic review [16] reported the then-current evidence (search dates until March 2016) of the diagnostic accuracy and validity of physical tests for patients with CR [16]. Preliminary scoping searches suggested that new studies had been published, warranting the need for an update. Additionally, most peer-reviewed journals publishing diagnostic accuracy studies encourage or expect adherence to reporting standards such as the QUADAS-2 and Standards for Reporting Diagnostic Accuracy (STARD) [21] guidelines, which were not used in this previous publication. Consequently, newer studies may demonstrate a reduced risk of bias compared to older studies, thereby influencing the certainty of the overall evidence.

Therefore, an update of the original systematic review [16] was conducted to answer the following question: “What is the diagnostic accuracy of physical tests used to assess for the presence of CR?” The risk of bias of the studies was assessed using the QUADAS-2 guidelines. To further increase the rigour and value of this current review with respect to the original review, the certainty of the evidence was also assessed using the Grading of Recommendations Assessment, Development and Evaluation (GRADE) method approach [22], which was not used in the original systematic review.

## Methods

This systematic review was registered in the PROSPERO database (CRD42023058465) in November 2023 and is written in compliance with the Preferred Reporting System and Meta-Analysis (PRISMA) 2020 guidelines [23]. All procedures adhered to the registered protocol; a GRADE assessment was added subsequently to evaluate the certainty of the evidence.

### Selection criteria

The PICOS framework, as endorsed by the Cochrane Collaboration, was used to develop the search strategy [24]. Studies were included if they assessed (P) patients suspected of having CR. The diagnosis of CR could be made clinically by an appropriate medical specialist and/or confirmed with medical imaging (MRI or CT). Eligible studies aimed to assess the diagnostic accuracy of (I) physical examination tests for identifying CR (i.e., how well a test, or a combination of tests, correctly identified patients with CR). The results of the index test(s) were compared (C) with a reference standard consisting of either (1) diagnostic imaging such as magnetic resonance imaging (MRI), computed tomography (CT) or myelography, or (2) findings during surgery [19, 25]. Studies that used electromyography (EMG) as the sole reference standard were excluded. Studies were eligible if they reported diagnostic accuracy outcomes (O), such as values of sensitivity, positive predictive value, specificity or negative predictive value. Primary (cross-sectional) studies conducted in both primary as well as secondary care were eligible (S).

### Exclusion criteria

Studies were excluded if full reports were unavailable (e.g., conference abstracts only) or if the outcome of interest was not reported. Case-control designs including healthy controls, were also excluded due to the high risk of bias and limited applicability. No language restrictions were applied during the initial screening.

### Search strategy

The initial systematic review [16] searched CENTRAL (The Cochrane Library Central Register of Controlled Trials), PubMed (including MEDLINE), Embase, CINAHL (Cumulative Index to Nursing and Allied Health Literature), Web of Science (WoS), and Google Scholar for eligible diagnostic studies from database inception to March 2016. The search strategy was developed in collaboration with a librarian and followed the guidelines set by the Cochrane Diagnostic Test Accuracy group.

For the present systematic review, the original search was adapted to the search interfaces of CINAHL, EMBASE, Medline, PubMed, WoS and Google Scholar, and executed with the support of an information specialist. All databases were searched from March 2016 to June 5th 2025. The results from both searches were combined. The detailed search strategies are listed as an Appendix (APPENDIX 1).

### Selection of studies

Retrieved records were imported into EndNote and, following duplicate removal, exported as an EndNote XML file and uploaded to Covidence (Veritas Health Innovation, Melbourne, Australia; available at www.covidence.org) to support screening and study selection. Two reviewers (ET, MA) independently screened titles and abstracts and subsequently assessed the full texts of potentially relevant articles for eligibility. Disagreements regarding inclusion were resolved through discussion and, where necessary, by arbitration from a third reviewer (DF).

### Data extraction

Characteristics of participants, the index tests and reference standard, and aspects of study methods for each included study were extracted by the two reviewers (ET, MA) using a standardised form identical to the form used in the original review. Diagnostic two-by-two tables (true positive [TP], false positive [FP], true negative [TN], and false negative [FN] positive likelihood ratios [LR+] and negative likelihood ratios [LR-]) for each study were also extracted or reconstructed if they were not available, using information on relevant parameters (e.g., sensitivity and specificity) as described by Shim et al., 2019 [26].

### Assessment of risk of bias

Risk of bias of each study was assessed independently by the same two reviewers (ET, MA) using the QUADAS-2 tool. Guidelines for the assessment of the four bias domains were provided to the reviewers to support consistent judgments (APPENDIX 2). For the QUADAS-2 domain relating to the reference standard, the identical tiered scoring system of the original review was used. A reference standard comprising a combination of history taking, physical examination, including neurological assessment and MRI or CT-myelography imaging (or surgical findings) was considered optimal and was rated “yes”. In contrast, a reference standard based on MRI or CT-myelography imaging alone was rated “unclear”, given the potentially high number of false-positive findings [27–29]. Incorporation bias was considered unlikely when the index test was not included with the reference standard. Inter-rater agreement was quantified using Cohen’s kappa *k* for the initial agreement on the overall score per domain [30]. Disagreements were resolved by consensus and, if necessary, through arbitration by the third reviewer (DF). QUADAS-2 assessments were summarised using both tabular and graphical displays.

### Statistical analysis

Two-by-two tables were constructed for each index test evaluated in each included study using the extracted number of TPs, FNs, TNs, and FPs. Study-level diagnostic accuracy outcomes (sensitivity, specificity LR+, LR-, diagnostic odds ratio; DOR) and their 95% confidence interval (CI) were calculated using IBM SPSS Statistics (Version 30).

When pooling across studies was possible, a hierarchical bivariate random-effects model was fitted using the glmer function from the lme4 package in R (version 4.4.3; R Development Core Team, 2023; lme4 version 1.1.37). This bivariate generalised linear mixed model (GLMM) is one of the two statistically rigorous approaches recommended by the Cochrane Handbook for Diagnostic Test Accuracy Reviews (alongside the hierarchical summary ROC; HSROC model) [31]. Because each index test was informed by a small number of studies (k ≤ 3), diagnostic checks revealed singular model fits, meaning that between-study variance components could not be reliably estimated [32, 33]. In line with recommendations for sparse data, the models were therefore interpreted as bivariate fixed-effect GLMMs, assuming homogeneous sensitivity and specificity across studies. These pooled estimates should be interpreted cautiously, given the limited evidence base. Pooled sensitivity and specificity were obtained from the model’s fixed effects, while DOR, LR+, and LR− were computed from these values. Confidence intervals for LR+, LR−, and DOR were calculated using the delta method, a standard approach that approximates uncertainty on the log scale before back-transforming to the original ratio scale [31, 33]. No continuity corrections were required. As shown in the individual study results (Table 3), none of the included values contained zero cell counts, the GLMM framework inherently accommodates such data without artificial adjustments, and the delta method was applied exclusively to pooled sensitivity and specificity estimates, which always lie strictly between 0 and 1, avoiding division-by-zero issues.

Individual study estimates contributing to each meta-analysis are presented visually in Paired Forest Plots (Fig. 4), constructed using the diagnostic accuracy values reported in Table 3. These plots allow readers to assess the consistency of study-level sensitivity and specificity. A summary ROC curve was not produced because the sparse data (k ≤ 3) did not allow meaningful estimation of random-effects variance or reliable SROC shape parameters. Instead, pooled results are illustrated in crosshair plots, displaying the pooled sensitivity and false positive rate (FPR = 1 − specificity) with their 95% CIs alongside individual study points. Sensitivity and specificity were classified as high (≥ 0.80), moderate (0.60–0.79), or low (< 0.60) [41].

To enhance clinical interpretability, Positive Predictive Values (PPV) and Negative Predictive Values (NPV) were calculated across four possible pre-test probabilities (5%, 15%, 30%, and 50%). In the absence of reliable externally validated estimates and with only one large cohort study from 1994 [42], only illustrative values are used. Post-test probabilities were subsequently derived from the pooled sensitivity (Sens), specificity (Spec), and each chosen pre-test probability (P) using the following standard formulas [43]:

Predictive values:

$$\:PPV=\:\frac{Sens\:\times\:\:P}{Sens\:\times\:\:P+\left(1-Spec\right)\times\:\:(1-P)}$$

$$\:NPV=\:\frac{Spec\:\times\:\:(1-P)}{(1-Sens)\:\times\:\:P+Spec\times\:\:(1-P)}$$

Conversion between probabilities and odds:

$$\:Pre-test\:odds=\:\frac{P}{1-P}$$

$$\:Post-test\:odds=Pre-test\:odds\:\times\:\:Likelihood\:Ratio$$

$$\:Post-test\:probability=\:\frac{Post-test\:odds}{Post-test\:odds+1}$$

These values were then used to generate Fagan nomograms for each diagnostic test, providing a visual representation of how baseline (pre-test) probabilities shift following positive or negative test results. This graphical approach illustrates the clinical impact of the pooled likelihood ratios and supports interpretation of whether each test meaningfully alters disease probability. All figures were generated in R (version 4.5.2) using the ggplot2 package (version 4.0.0) [44].

### Certainty of the evidence

The certainty of the evidence was assessed using the GRADE approach [45]. According to this approach, the certainty of the evidence is determined across five domains: study limitations (risk of bias), inconsistency, indirectness, imprecision, and publication bias. The certainty of the evidence was downgraded by one level when concerns were identified in any domain, resulting in an overall certainty rating for each outcome (e.g., sensitivity, specificity) of high, moderate, low, or very low. The certainty of the evidence was initially rated as ‘high’ and then was downgraded as appropriate based on the criteria above. Evidence derived from single studies was considered inconsistent and imprecise (i.e., sparse data) and was therefore rated as “low certainty evidence’’, with further downgrading to ‘‘very low certainty evidence’’ when additional concerns were present (e.g., risk of bias or indirectness). Based on preliminary scoping searches indicating that few randomised controlled trials would be available, upgrading of certainty was not anticipated.

## Results

### Search results

**Fig. 1:**
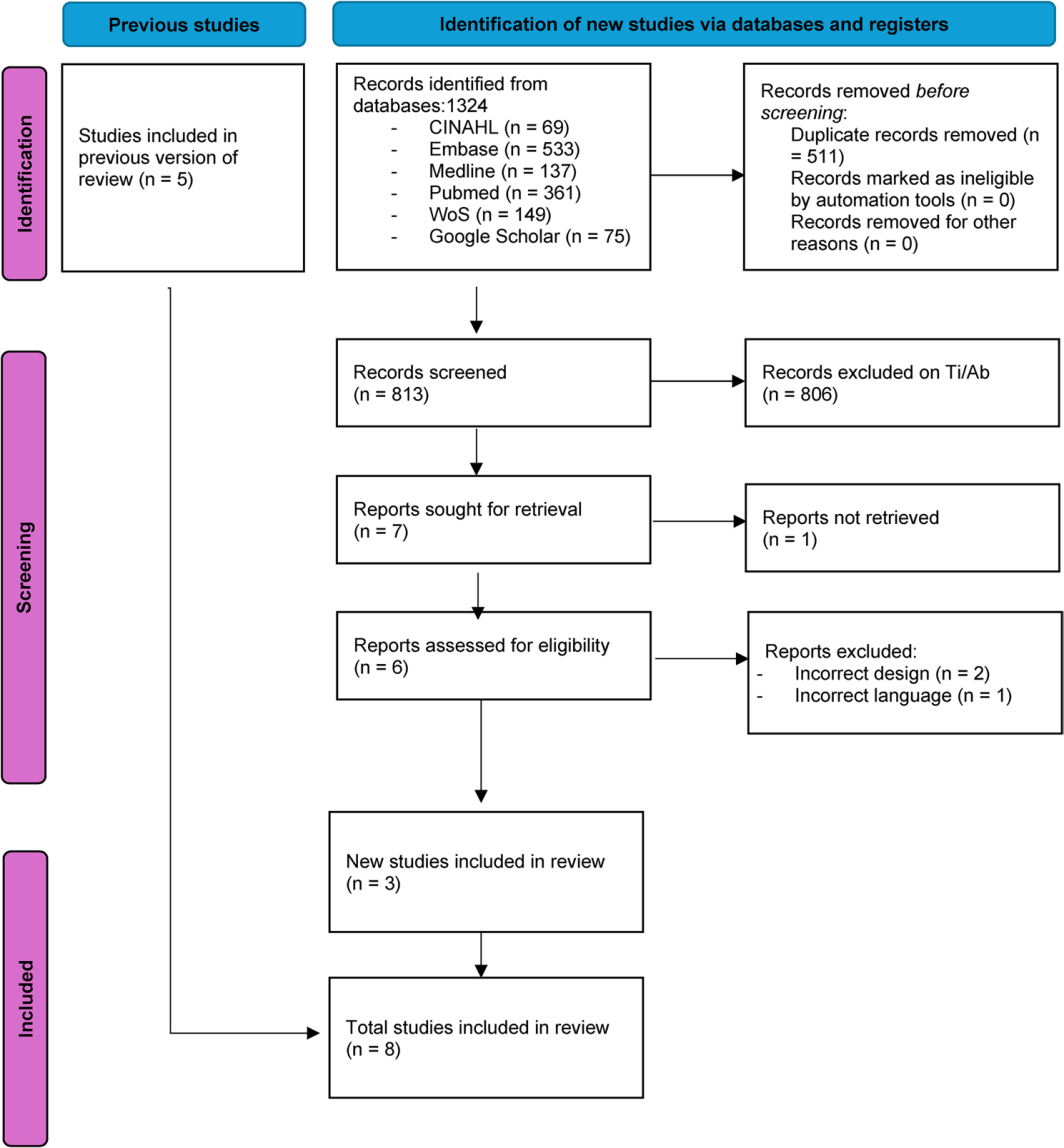
PRISMA flow chart of included studies

The search identified 1.324 studies, which were then selected for full-text assessment based on title and abstract screening (Fig. 1, PRISMA Flow chart [23]). One study was excluded based on the authors’ inability to fully comprehend the study due to (Italian) language restrictions. Three new studies (not reported in the Thoomes, 2018 review) matching the predefined PICO eligibility criteria were included in the assessment [39–41], which makes the final number of included studies eight. These three studies are summarised below. The prospective cohort study by Grondin [39] tested the diagnostic accuracy of single and combined upper limb neurodynamic tests (ULNTs) and included 85 patients between September 2017 and September 2019 [39]. The retrospective study by Park [40] reviewed records of 135 patients who were referred to a pain clinic between September 2014 and August 2015 and assessed the diagnostic accuracy of the Spurling test and the newly introduced ‘neck tornado test’ (NTT or ‘Choi’s test’) [40]. The prospective cohort study by Sleijser-Koehorst [41] assessed the diagnostic accuracy of the Spurling test, ULNT1 test and the shoulder abduction test in 134 patients in an unspecified time frame [41]. The characteristics of all eight included studies are described in Table 1.

**Table 1:** Characteristics of the included studies

Study	Characteristics		Diagnostics			Study design
	Setting	Population	Indextest	Comparator test	Reference test (cut-off)	
Apelby-Albrecht [34]	Center for Spinal surgery Country: Sweden Prevalence: 0.69 (95% CI 0.54 to 0.81)	*Mean age*: NR *Female (%)*: NR *Duration of pain*: NR	ULNT1 (median), ULNT2a (median), ULNT2b (radial) and ULNT3 (ulnar)	-	1: Clinical examination, medical history and; 2: MRI-scan and; 3 history	Diagnostic cohort study
Gumina [35]	Shoulder Clinical Office and Orthopedic Spine Ambulatory Country: Italy Prevalence: 0.20 (95% CI 0.18 to 0.22)	*Median age*: NR *Female (%)*: NR *Duration of pain*: NR	Arm squeeze test	-	1: Clinical examination and; 2: MRI-scan and; 3 history	Cohort study
Shabat [36]	Spine Surgery Unit Country: Israel Prevalence: 0.68 (95% CI 0.71 to 0.75)	*Median age*: NR *Female (%)*: NR *Duration of pain*: NR	Spurling (Ext + Rot+Ax compression)	-	Complete physical examination and MRI/CT imaging	Cohort study
Shah [37]	Neurosurgical Unit Country: India Prevalence: 0.86 (95% CI 0.72 to 0.82)	*Mean age*: NR *Female (%)*: NR *Duration of pain*: NR	Spurling (Ext + LF+Ax pressure)	-	T-2 weighted axial MRI	Prospective cohort study
Viikari-Juntura [38]	Neurosurgery department Country: Finland Prevalence:	*Mean age*: NR *Female (%)*: NR *Duration of pain*: NR	Spurling (LF + Rot+Ax compression) Shoulder abduction relief test Axial traction test	-	1: conventional neurological examination and; 2: Cervical myelography	Prospective cohort study
Grondin [39]	Neurosurgery department Country: France Prevalence: 0.317	*Mean age (SD)*: 44 (CR+) and 45 (CR-) *Female (%)*: NR *Duration of pain*,* months (SD)*: 93 (98) for CR + and 71 (62) for CR-	ULNT1 (median), ULNT2a (median), ULNT2b (radial) and ULNT3 (ulnar)	-	1: diagnosis based on clinical presentation by neurosurgeon and; 2: MRI-scan	Prospective cohort study
Park [40]	Pain clinic in hospital Country: Korea Prevalence: 0.50 (95% CI 0.41 to 0.58)	*Mean age*: 53.4 (13.1) *Female (%)*: 57 (42) *Duration of pain*: NR	Spurling (Ext + Rot+Ax pressure)		1 diagnosis based on clinical presentation by neurosurgeon and; 2: MRI-scan	Retrospective study
Sleijser-Koehorst [41]	Multidisciplinary clinic Country: the Netherlands Prevalence: 0.37 (0.27 to 0.48)	*Mean age (SD)*: 49.9 (10.7) *Female (%)*: 65 (48.5) *Median duration of pain*,* weeks (IQR)*: 26 (13–104)	Spurling (Ext + Rot+LF)	ULNT1, Shoulder abduction test, and cervical distraction test	1: diagnosis based on clinical presentation by neurosurgeon and; 2: MRI-scan	Prospective cohort study

*Abbreviations*: *Ax* Axial compression/pressure, *CR*+ Subjects with cervical radiculopathy, *CR* Subjects without cervical radiculopathy, *Ext* Extension, *LF* Lateral flexion, *NR* Not reported, *Rot* Rotation, *SD* Standard deviation, *ULNT* Upper Limb Neurodynamic Tests, "prevalence” refers to the calculated prevalence of CR in the study

### Risk of bias

**Fig. 2:**
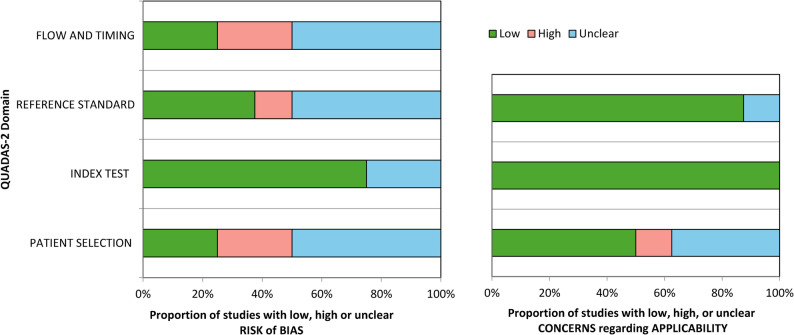
QUADAS-2. Proportion of studies with low, high or unclear risk of bias

The risk of bias of the three newly included studies was lower than that of most of the studies originally included (see Table 2; Fig. 2). Inter-rater agreement across all included studies was almost perfect (*k =* 0.88, *p* < 0.001), and arbitration through the third reviewer was not required. Two studies were considered to be of low risk of bias [39, 41].

**Table 2:** Tabular presentation for QUADAS-2 results

Study	Risk of bias				Applicability concerns		
	Patient selection	Index test	Reference standard	Flow and timing	Patient selection	Index test	Reference standard
Apelby-Albrecht [34]	Unclear	Low	Low	High	Low	Low	Low
Gumina [35]	High	Low	Unclear	Unclear	Unclear	Low	Low
Shabat [36]	Unclear	Unclear	Unclear	Unclear	Unclear	Low	Unclear
Shah [37]	Unclear	Unclear	Unclear	Unclear	Unclear	Low	Low
Viikari-Juntura [38]	High	Low	Unclear	High	High	Low	Low
Park [40]	Unclear	Low	High	Unclear	Unclear	Low	Low
Grondin [39]	Low	Low	Low	Low	Low	Low	Low
Sleijser-Koehorst [41]	Low	Low	Low	Low	Low	Low	Low

For the patient selection domain, two of the eight included studies were rated as having high risk of bias: one study [35] strongly resembled a case control study and one study [38] had inappropriate exclusion criteria. Two studies were classified as ‘unclear’ risk of bias in the patient selection domain: one study [40] was a retrospective study, and in one study, patients were referred to a multidisciplinary clinic for conservative management, where one would expect that consideration for surgery would be the reason for referral [41]. Regarding the applicability to the review question, one study [38] had serious concerns due to an unclear process for excluding patients or which tests had been conducted prior to inclusion in the study, as exclusions seemed likely to have taken place after history taking and the physical examination. This does not reflect the intended use of the index test. Three studies were unclear in this domain [35, 36, 40].

For the index test domain, no studies were rated as having high risk of bias. Two studies [36, 37] were classified as “unclear” because it was not clear if the index test results were interpreted without knowledge of the results of the reference standard. Six studies [34–36, 38, 39, 41] specified a positivity threshold (interpretation of “positive” results). No concerns were identified regarding the applicability of any of the studies.

With respect to the reference standard, three studies [34, 39, 41] were considered to have an appropriate reference test (low risk of bias), and one study assessed the root canal diameter on MRI for all patients, and for a portion of patients, the results at surgery [37]. The remaining studies did not include information on the type of physical examination with the information in their (MRI or CT-myelography) reference standard conclusion, or were unclear with respect to blinding of assessors, resulting in an unclear score [35–37]. In one study, the reference standard results were interpreted with knowledge of the results of the index test [40].

The most common concerns were related to patient flow and timing. Two studies used different reference tests for some patients [36, 37]. One study [38] had too many missing patients, and not all included patients received the same reference standard or index test, while another study [34] reported an inappropriate time between the reference and index test. In one retrospective study [40], it was not clear if the index test results were interpreted without knowledge of the results of the reference standard. Other studies did not report on the time between the reference and index test.

### Certainty of the evidence

The justification for downgrading the certainty of the evidence for the different diagnostic tests is presented in the GRADE Summary of Findings tables (APPENDIX 3).

### Diagnostic accuracy

Diagnostic accuracy was assessed for the following physical tests: Spurling’s test, ULNT’s, shoulder abduction test, arm squeeze test, axial traction test and neck tornado test. The reported or recalculated estimates of diagnostic accuracy of each of the tests for the individual studies are presented in Table 3. The ULNT1, the combined ULNTs and the shoulder abduction test were sufficiently homogenous to allow pooling of results and are highlighted in Table 3.

**Table 3:**
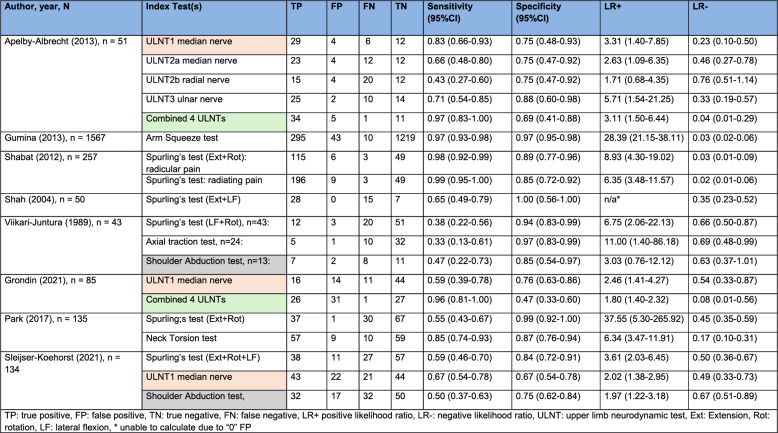
Diagnostic accuracy of the included studies. The individual studies that contributed to each meta-analysis have been highlighted to aid interpretation

#### Spurling’s test

Spurling’s test was evaluated in five studies, but all studies used slightly different ways of executing the test or interpreting the results (see Table 3) [37, 40, 41]. It is therefore difficult to compare the different outcomes. There is low certainty evidence of specificity ranging from 0.84 to 1.00 (95% CI range: 0.56–1.00). There is very low certainty evidence of sensitivity ranging from “low” to “high” with values ranging from 0.38 to 0.98 (95%CI range: 0.22–0.99).

There is very low certainty evidence of LR + and LR- of Spurling’s test for diagnosing CR [37, 40, 41].

#### Upper limb neural tension tests

**Fig. 3:**
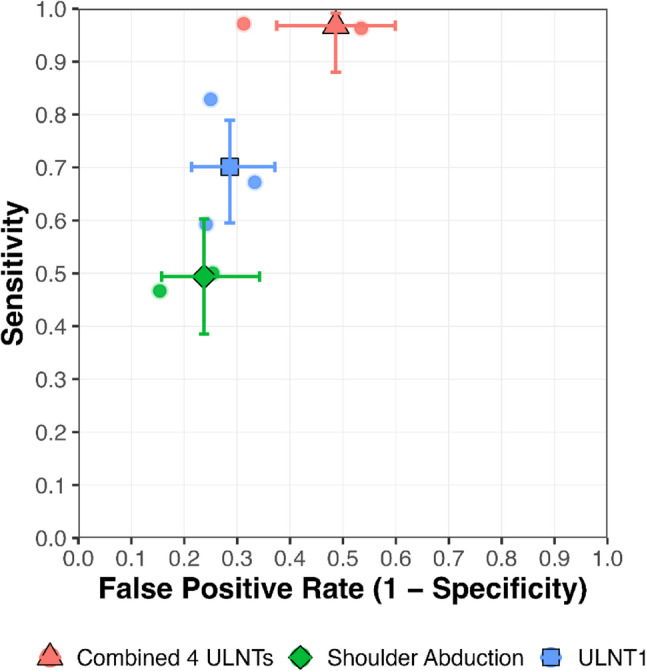
Crosshair plot of diagnostic test accuracy meta-analysis. The plot shows pooled sensitivity (y-axis) against false positive rate (1 − specificity, x-axis) for each index test. Individual study results appear as smaller dots in the same colour as their corresponding test. Large solid symbols (triangle = combined 4 ULNTs, diamond = shoulder abduction test, square = ULNT1) indicate pooled estimates from the bivariate GLMM. Vertical and horizontal bars represent 95% confidence intervals for sensitivity and false positive rate. Colours correspond to test type: red = combined 4 ULNTs, green = shoulder abduction test, blue = ULNT1. ULNT: upper limb neurodynamic test

**Fig. 4:**
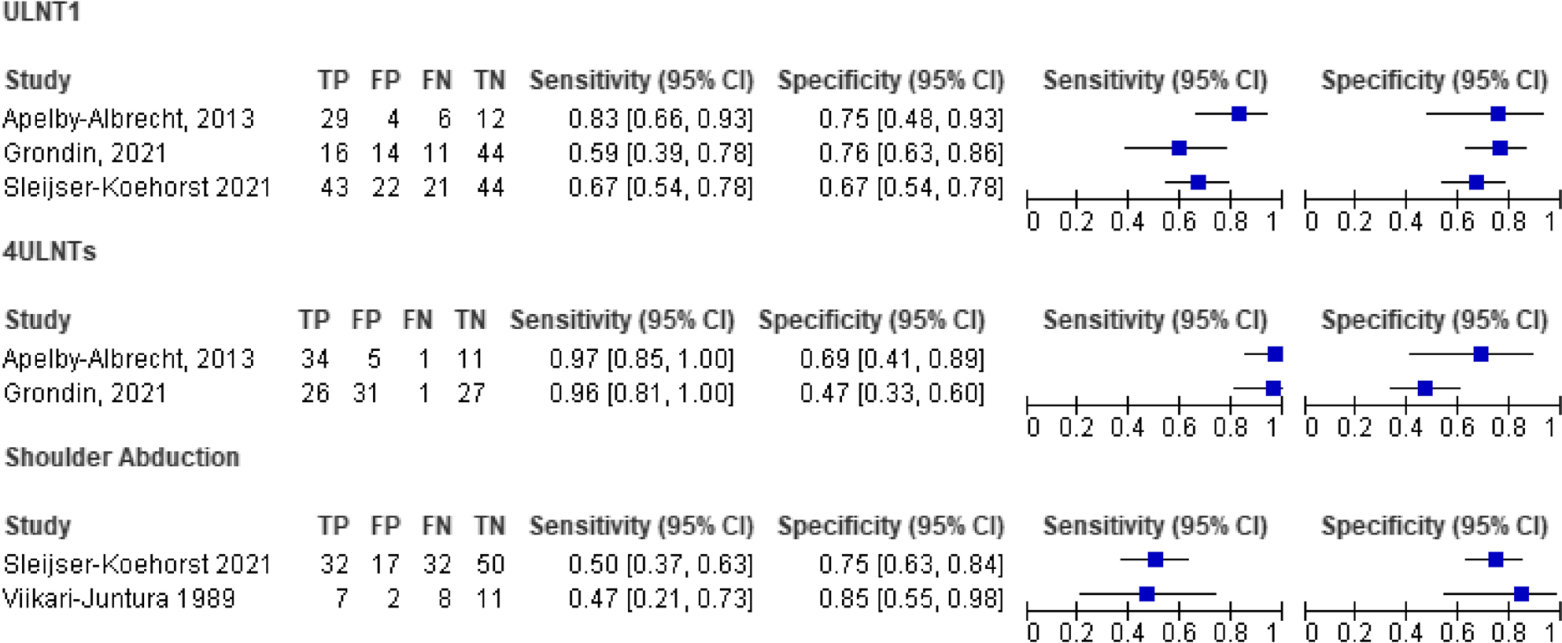
Paired forest plots of sensitivity and specificity for the studies included in the meta-analysis. The studies displayed in each panel correspond to those contributing to the pooled diagnostic estimates for that specific test, allowing visual comparison of their individual performance. ULNT = Upper Limb Neurodynamic Test

Three recent studies assessed the diagnostic value of the ULNT 1 (with a median nerve bias) [34, 39, 41]. As both the execution and the cut-off point of the ULNT1 in these studies were similar, the data from these three studies were pooled, resulting in very low certainty evidence of pooled sensitivity for ULNT1 of 0.70 (95%CI 0.60–0.79) and specificity of 0.71 (95%CI 0.63–0.79), DOR of 5.85 (95%CI 3.16–10.84), LR + of 2.45 (95%CI 1.79–3.36) and LR- of 0.42 (95%CI 0.30–0.59) (see Figs. 3and 4; Table 4).

**Table 4:** Results of meta-analysis of measures of diagnostic performance using a bivariate fixed-effect GLMM

Index Test	Sensitivity (95%CI)	Specificity (95%CI)	LR+ (95%CI)	LR- (95%CI)	DOR (95%CI)
ULNT1 alone	0.70 (0.60–0.79)	0.71 (0.63–0.79)	2.45 (1.79–3.36)	0.42 (0.30–0.59)	5.85 (3.16–10.84)
Combined ULNTs; 1 positive	0.97 (0.88–0.99)	0.51 (0.40–0.62)	1.99 (1.57–2.52)	0.06 (0.02–0.25)	31.67 (7.20–139.21)
Shoulder Abduction test	0.49 (0.39–0.60)	0.76 (0.66–0.84)	2.08 (1.32–3.27)	0.66 (0.52–0.85)	3.13 (1.59–6.17)

*LR*+ positive likelihood ratio, *LR*- negative likelihood ratio, *DOR* Diagnostic odds ratio, *ULNT* Upper limb neurodynamic test

Recently, two studies also reported the diagnostic accuracy of using a combination of four ULNTs [34, 39]. There is very low certainty evidence of pooled sensitivity for combined ULNTs of 0.97 (95%CI 0.88–0.99) and pooled specificity of 0.51 (95%CI 0.40–0.62), DOR of 31.67 (95%CI (7.20–139.21), LR + of 1.99 (95%CI 1.57–2.52) and LR- of 0.06 (95%CI 0.02–0.25) (see Figs. 3 and 4; Table 4).

#### Shoulder abduction test

Two studies reported on the shoulder abduction test [38, 41]. There is very low certainty evidence of pooled sensitivity of the shoulder abduction test of: 0.49 (95%CI 0.39–0.60) and pooled specificity of 0.76 (95%CI 0.66–0.84), DOR of 3.13 (95%CI 1.59–6.17), LR + of 2.08 (95%CI 1.32–3.27) and LR- of 0.66 (95%CI 0.52–0.85) (see Figs. 3 and 4; Table 4).

#### Arm squeeze test, traction test and neck tornado test

These tests were all assessed in one single study only [38, 40]. There is very low certainty evidence of high sensitivity of 0.97 (95%CI 0.93–0.98) and high specificity of 0.97 (95%CI 0.95–0.98) for the arm squeeze test for diagnosing CR [35]. There is very low certainty evidence of low sensitivity 0.33 (95%CI 0.13–0.61), and high specificity 0.97 (95%CI 0.83–0.99) of the axial traction test for diagnosing CR [38]. There is very low certainty evidence of high sensitivity 0.85 (0.74–0.93) and specificity 0.87 (0.76–0.94) of the neck tornado test in diagnosing CR [40] (see Table 3).

Inspection of the paired forest plots (Fig. 4) revealed that the combined four ULNTs showed the highest consistency, particularly for sensitivity, where estimates clustered tightly with overlapping confidence intervals. ULNT1 demonstrated moderate variability across studies, with overlapping but wider CIs for both sensitivity and specificity. In contrast, Shoulder Abduction exhibited the greatest between-study heterogeneity, as reflected in the broad spread and minimal overlap of CIs. These patterns are consistent with the limited number of available studies and highlight the need for additional high-quality diagnostic research in this area.

### Clinical relevance

To enhance the clinical decision-making utility of the findings, a Clinical Utility Table (Table 5) was generated, presenting PPV and NPV across a range of potential prevalences (5%, 15%, 30%, and 50%). This illustrates PPV and NPV vary as a function of the underlying pre-test probability [43].

**Table 5:** Clinical utility table

Test	Prevalence_Pct	PPV	NPV
Combined 4 ULNTs	5%	9.5%	99.7%
Combined 4 ULNTs	15%	26.0%	98.9%
Combined 4 ULNTs	30%	46.0%	97.4%
Combined 4 ULNTs	50%	66.5%	94.1%
ULNT1	5%	11.4%	97.8%
ULNT1	15%	30.2%	93.1%
ULNT1	30%	51.2%	84.8%
ULNT1	50%	71.0%	70.5%
Shoulder Abduction	5%	9.9%	96.6%
Shoulder Abduction	15%	26.8%	89.5%
Shoulder Abduction	30%	47.1%	77.8%
Shoulder Abduction	50%	67.5%	60.1%

*ULNT* Upper limb neurodynamic test. Note: In the absence of reliable externally validated estimates, only illustrative values are used

**Fig. 5:**
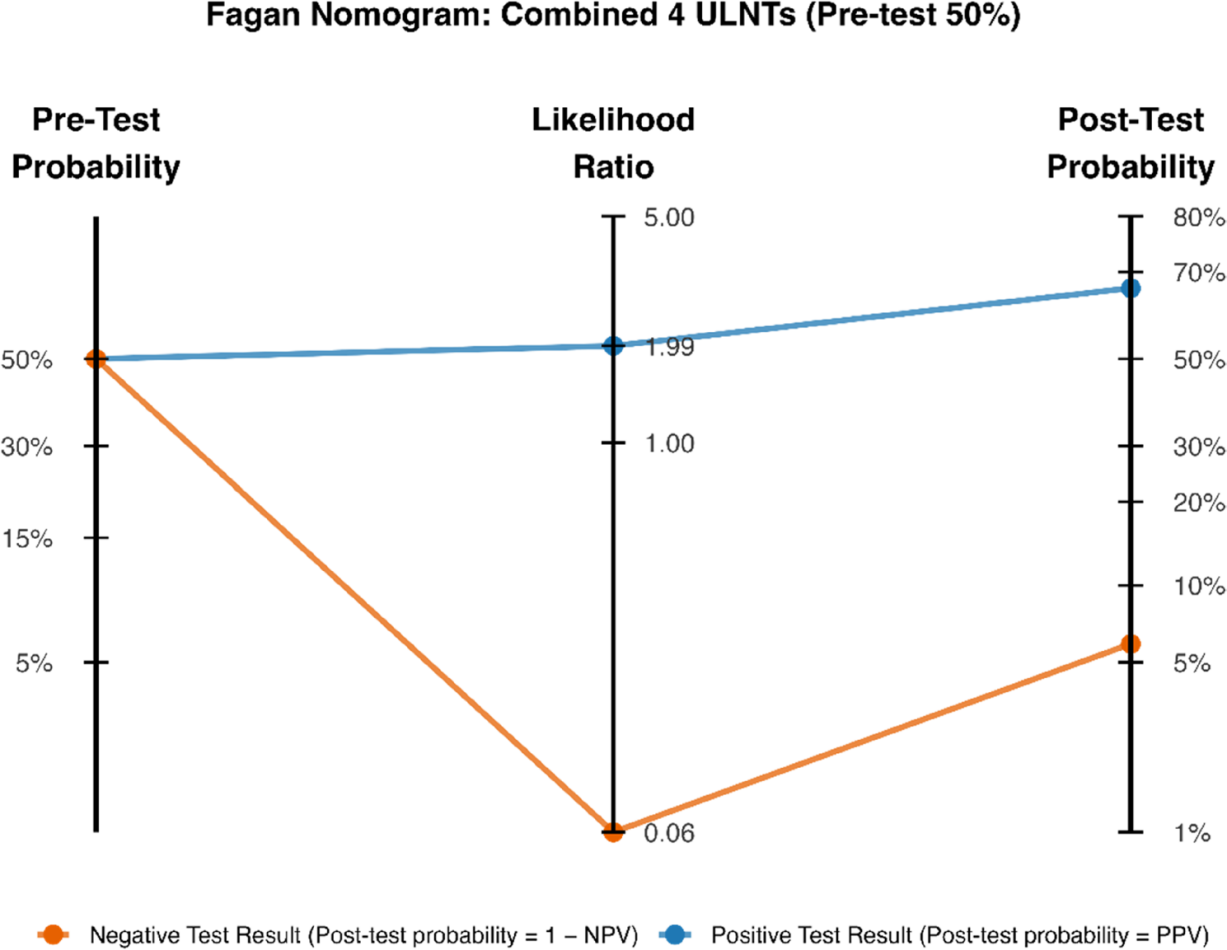
Fagan nomogram for the combined four ULNTs at a pre-test probability of 50%. This nomogram illustrates how the pooled likelihood ratios for the Combined four Upper Limb Neurodynamic Tests (ULNTs) translate a 50% pre-test probability of neuropathic pain into post-test probabilities following a positive or negative test result. The blue line represents a positive test result (LR⁺ = 1.99), increasing the probability of neuropathic pain from 50% to approximately 67%. The orange line represents a negative test result (LR⁻ = 0.06), reducing the probability from 50% to approximately 6%. Pre-test probability, likelihood ratios, and post-test probability are shown on the three aligned axes. This visualisation demonstrates the strong rule-out value of the Combined 4 ULNTs when the result is negative.ULNT: upper limb neurodynamic test

Furthermore, Fagan Nomograms [46] were included for each type of test across a range of clinically plausible prevalences (5%, 15%, 30%, and 50%) (APPENDIX 4) to visually demonstrate the change from pre-test to post-test probabilities, and a brief worked example was added (Fig. 5) to assist clinicians in interpreting these findings.

## Discussion

This systematic review and meta-analysis aimed to assess the value of physical examination tests used to diagnose CR by updating a previously published systematic review [16]. Three additional studies were identified and included, and the GRADE approach was applied to rate the certainty of the evidence across all outcomes [22]. Compared to the original review, additional evidence is now available for the diagnostic accuracy of Spurling’s test, the ULNTs and the shoulder abduction test. The update also incorporated one newly proposed test, the Neck Torsion test [40].

A previous systematic review concluded that (when consistent with the history and other physical findings), a positive Spurling’s, traction test and Valsalva’s manoeuvre might be indicative of CR, but that a negative ULNT might be used to rule it out [15]. With respect to Spurling’s test, the present review confirmed this high specificity, which was also mentioned in the original systematic review [16]. The current review also showed that the sensitivity of the ULNT1 ranged from moderate to high, confirming that a negative ULNT1 might assist in ruling a CR out. In addition, the present study showed the high sensitivity of having one test positive in performing a cluster of four ULNTs. One study on the diagnostic value of Valsalva’s manoeuvre [17] was not added to the present review, as that study used a different reference test (i.e., EMG) than the reference test proposed in the inclusion criteria.

The moderate specificity of 0.71 (95% CI 0.63–0.79) and moderate sensitivity of 0.70 (95% CI 0.60–0.79), reported in the present review are consistent with the results of a recent systematic review evalulating the diagnostic accuracy of ULNT1 alone, which reported a pooled specificity of 0.54 (95% CI 0.36–0.71) and sensitivity 0.69 (95% CI 0.50–0.83) and a summary receiver operating characteristic curve area of 0.65 (95% CI 0.61–0.69) [47]. As expected, the criterion of one positive test out of four ULNTs tests was the most sensitive combination and, as such, has the ability to “rule out” CR as shown in the study by Grondin et al. [39], which reported a negative LR of 0.08 (or strong evidence [48, 49]). Additionally, the condition of having four out of four ULNT tests positive was the most specific and has been reported to have an infinite LR + in the study by Grondin et al. [39]. However, it should be noted that in that study, there were only three cases in which all four tests were positive. The condition of having three out of four tests positive was reported to have a positive LR of 12.89 (‘strong evidence’ [48, 49]).

A recent systematic review assessed the diagnostic accuracy of ULNTs and their variations and criteria for upper-limb entrapment neuropathies, including CR and carpal tunnel syndrome [50]. Because that review included studies using EMG as a reference standard, it incorporated two additional studies [17, 51] that were excluded from the present review. This difference in eligibility criteria may explain the slightly different estimates reported for ULNT1 of 0.73 (0.48–0.88) and specificity of 0.52 (0.35–0.69) compared with the present findings (0.70 (0.60–0.79) and 0.71 (0.63–0.79), respectively). The authors, however, mention that only the study of Apelby-Albrecht et al. [34] reports on a combination of ULNTs whereas the study of Grondin et al. [39] (which they also include) also does so.

### Strengths and limitations

The present systematic review has several strengths in that it followed the guidelines set by the Cochrane Diagnostic Test Accuracy group, assessed the risk of bias of all studies using the QUADAS-2 guidelines and assessed the certainty of the evidence using the GRADE approach. Nevertheless, there are some limitations which need to be acknowledged. Cohen’s kappa for interrater reliability during the initial inclusion process was not calculated. Additionally, the exclusion of one study due to language restrictions might potentially have led to a small publication bias. A further important limitation is the small number of studies available for each index test (k ≤ 3). This sparsity resulted in singular model fits in the hierarchical bivariate GLMM, indicating that the between-study variance or heterogeneity could not be reliably estimated. Consequently, because of the small number of studies per test, fixed-effect models were used, and the models were interpreted as fixed-effect GLMMs, which assume homogeneity across studies. In contrast to random-effects models, fixed-effect models assume a single common true accuracy shared by all included studies and do not support extrapolation to a broader population. The pooled estimates are therefore valid only for the populations and tests studied in the specific studies included in this review. They cannot be reliably generalised to other settings, populations, or implementations of the tests that differ from those represented in these few studies. Therefore, the pooled estimates should be interpreted with caution. Similarly, the clinical translation analyses (PPV/NPV tables and Fagan nomograms) were derived from these same limited pooled estimates. Although these tools provide useful decision-making context, their clinical applicability is restricted by the small evidence base.

Following contemporary guidelines [18–20], EMG was deemed not to be a valid comparator or reference test, as it suggested it would only be positive in case of axonal damage. These studies were therefore excluded, but this might have led to potentially missing studies with valuable information.

The evidence on the diagnostic accuracy of physical tests for diagnosing CR is still sparse. Additionally, the certainty of the evidence is very low. This was mainly due to methodological shortcomings (risk of bias) and broad confidence intervals, often combined with small study populations (imprecision) and clinical heterogeneity. Therefore, based on the available literature, no solid conclusions can be drawn.

### Clinical implications

A best evidence synthesis based on the results from this systematic review suggests that a positive Spurling’s test combined with a positive result on a four-test ULNT cluster increases the likelihood of a diagnosis of CR. Incorporating the outcome of the Arm Squeeze test (due to the large cohort it was studied in) and the Shoulder Abduction test (as it was assessed in > 1 study) might be used to further increase this likelihood. Negative outcomes of the Spurling’s test and also the outcome of a cluster of four ULNTs can increase the likelihood of ruling out CR.

Nevertheless, all included studies were performed in secondary health care settings, limiting applicability to primary care settings. Patients visiting secondary care settings potentially have more serious or disabling complaints than those being referred to or seen in primary care settings.

CR is usually proposed as a clinical diagnosis [15, 16, 19, 20, 52]. As such, the use of the ULNTs for CR is similar to the use of the Straight Leg Raise or Lasègue’s test in lumbo-sacral radiculopathy [53], where the Straight Leg Raise is also a test of high sensitivity used mainly to rule the condition out [54, 55].

A complete patient history taking leading to the hypothesis of CR is one of the most important diagnostic tools for any clinician [56, 57]. Augmenting the diagnostic probability from that hypothesis with physical examination tests is a cost-effective way when compared to diagnostic imaging, such as MRI or CT. Additionally, these tests have been proposed to be used only in patients failing conservative management and being potential candidates for surgery [19, 20, 58].

### Implications for future research

As only one recent study [41] evaluated elements of the neurological bedside examination and patient history, future research should focus on establishing the validity and diagnostic accuracy of the neurological examination (myotendon reflexes, sensibility testing and key muscle strength testing) commonly used by both General Practitioners and medical specialists (neurologists, neurosurgeons) when assessing patients with radiating arm pain. Additionally, further work is needed to determine the most valid and reliable method for performing Spurling’s test, given the substantial variation in its execution across studies [59]. The diagnostic accuracy specific patient interview items warrants further investigation [41].

The diagnostic value of neurodynamic testing raises the question of whether neurodynamic mobility is altered in patients with CR. One study assessed the longitudinal excursion of the median nerve during a mechanically induced contralateral cervical lateral glide movement in patients with a CR and compared the findings with those of healthy matched controls [60]. The authors reported a significant between-group difference at baseline, which was no longer evident at three months’ follow-up [60].

The very low certainty of evidence on the proposed physical examination tests may indicate the need for alternative diagnostic approaches. Recently, a study assessed the reliability of a mechanism-based clinical framework for spine-related pain, classifying presentations into: (1) somatic referred pain, (2) heightened nerve mechanosensitivity, (3) radicular pain, (4) radiculopathy and (5) mixed-pain [5]. Substantial to almost perfect intra-rater reliability was reported for radicular pain (0.76 (95% CI: 0.57–0.92)), radiculopathy (0.84 (95% CI: 0.67–0.96)) and the overall framework (0.80 (95% CI: 0.61–0.92)) [5]. Further research is required to establish the validity of this classification system.

## Conclusion

Clinicians may use the outcome of Spurling’s test and the outcome of the combined four ULNTs to assist their clinical reasoning as an adjunct to history taking and bedside neurological examination when diagnosing CR. However, evidence on the diagnostic accuracy of all physical examination tests for the diagnosis of CR is sparse, and the certainty of the evidence was very low for all outcomes of all tests, implying that new studies of high methodological value are required to strengthen these results. Because of the small number of studies, the pooled estimates are therefore valid only for the specific studies included in this review and cannot be reliably generalised to other settings, populations, or implementations of the tests that differ from those represented in these studies.

## Supplementary Information

Supplementary Material 1.

Supplementary Material 2.

Supplementary Material 3.

Supplementary Material 4.

## Data Availability

The datasets used and/or analysed during the current study are available from the corresponding author on reasonable request.

## Abbreviations

Ax: Axial compression/pressure
CI: Confidence Interval
CINAHL: Cumulative Index to Nursing and Allied Health Literature
CR: Cervical Radiculopathy
CT: Computer Tomography
DOR: Diagnostic Odds Ratio
EMG: Electro Myography
Ext: Extension
FN: False Negative
FP: False Positive
FPR: False Positive Rate
GRADE: Grading of Recommendations Assessment, Development and Evaluation
LF: Lateral flexion
LR-: Negative Likelihood Ratio
LR+: Positive Likelihood Ratio
MRI: Magnetic Resonance Imaging
NR: Not reported
NTT: Neck Tornado Test
PRISMA: Preferred Reporting System and Meta-Analysis
QUADAS-2: Quality Assessment of Diagnostic Accuracy Studies
ROC: Receiver Operating Characteristic
Rot: Rotation
SD: Standard deviation
STARD: Standards for Reporting Diagnostic Accuracy
TN: True negative
TP: True Positive
ULNT: Upper Limb Neurodynamic Test
WoS: Web of Science
