# Supplementary material for: Diagnostic accuracy of physical examination tests for painful cervical radiculopathy: update of a systematic review and meta-analysis
Source: BMC Musculoskelet Disord. 2026 Feb 13;27:338. doi: 10.1186/s12891-026-09551-0 (PMC13088722; doi:10.1186/s12891-026-09551-0)
Supplement: Supplementary file 1 — Supplementary Material 1. [file 12891_2026_9551_MOESM1_ESM.docx]

**APPENDIX 1 SEARCH STRATEGIES**

**MEDLINE Search Strategy: Cervical Radiculopathy Physical Examination Tests**

Search Strategy Overview

Database: MEDLINE (Ovid Interface)

Date Range: March 1, 2016 - June 5, 2025

Document Types: Exclude Comments, Editorials, Letters

Language: English

Population: Human studies only

________________________________________

Individual Search Sets

#1 - Condition (Cervical Radiculopathy)

1. exp Radiculopathy/ or Brachial Plexus Neuropathies/ or Brachial Plexus Neuritis/ or cervicobrachial*.ti,ab,kf. or "cervico brachial*".ti,ab,kf. or "cervical brachial*".ti,ab,kf. or ((cervic* or brachial*) adj3 (neuralg* or compress* or radiculop* or avulsion* or radiculitis* or radiculitides* or syndrome* or myelopath* or spondylos* or osteophytos* or stenosis* or degenerat* or neuritis*)).ti,ab,kf. or ((radiculalgia or radiculitis or radiculitides or radiculopath* or polyradiculopath* or neuralgia or "herniated disc*" or hernia or (radicular adj3 (pain* or neuralgia* or symptom* or syndrom*)) or ("nerve root" adj3 (pain* or inflammation* or disorder* or compression* or avulsion* or impingement))).ti,ab,kf. and (exp Cervical Vertebrae/ or exp Neck/ or cervical.ti,ab,kf. or cervico*.ti,ab,kf. or neck.ti,ab,kf.)) or ((Spinal Cord Compression/ or Intervertebral Disc Displacement/ or Spinal Stenosis/ or Intervertebral Disc Degeneration/ or Spondylosis/) and (exp Neck/ or Neck Pain/ or Neck Injuries/ or exp Cervical Vertebrae/ or Cervical Plexus/ or exp Spinal Injuries/ or Cervical Cord/ or exp Spinal Cord Injuries/))

#2 - Physical Examination/Tests

2. Physical Examination/ OR Physical Therapy Specialty/ OR Physical Therapists/ OR exp Neurologic Examination/ OR physiotherap*.ti,ab,kf. OR (medical* adj3 examin*).ti,ab,kf. OR provocat*.ti,ab,kf. OR movement*.ti,ab,kf. OR abduction*.ti,ab,kf. OR spurling*.ti,ab,kf. OR "arm squeeze test*".ti,ab,kf. OR wainner.ti,ab,kf. OR (test adj3 cluster).ti,ab,kf. OR "neck tornado test*".ti,ab,kf. OR (tendon* adj3 reflex*).ti,ab,kf. OR manipulat*.ti,ab,kf. OR ((manual OR physical* OR neurodynamic OR "neuro dynamic" OR neurolog*) adj3 (measur* OR assess* OR rating* OR test* OR exam*)).ti,ab,kf. OR (((sensor* OR sensat*) adj3 (impair* OR dysfunction* OR abnormal*)).ti,ab,kf.) OR valsalva* maneuv*.ti,ab,kf. OR (((elvey OR davidson* OR distraction* OR relief* OR tension OR motor OR "upper limb" OR "brachial plexus") adj3 test*).ti,ab,kf.) OR ultt*.ti,ab,kf. OR ("shoulder abduction" adj3 (sign OR test*)).ti,ab,kf.

#3 - Diagnostic Accuracy Terms

3. exp "Sensitivity and Specificity"/ or (sensitivity or sensitive or specificity).ti,ab. or (ROC-curve or receiver-operator*).ti,ab. or (likelihood or LR*).ti,ab. or exp Diagnostic Errors/ or (inter-observer or intra-observer or interobserver or intraobserver or validity or kappa or reliability).ti,ab. or reproducibility.ti,ab. or (test adj2 (re-test or retest)).ti,ab. or "Reproducibility of Results"/ or accuracy.ti,ab. or Diagnosis, Differential/ or Validation Studies.pt. or exp "Predictive Value of Tests"/ or ppv.ti,ab,kf. or npv.ti,ab,kf. or ((false or true) adj3 (negative or positive)).ti,ab.

#4 - Combined Core Search with Filters

4. 1 and 2 and 3 and 20160301:20250605.(dt). not (comment/ or editorial/ or letter/) not ((exp animals/ or exp models, animal/) not humans/)

#5 - Systematic Reviews

5. meta-analysis/ or meta-analysis as topic/ or (metaanaly* or meta-analy* or metanaly*).ti,ab,kf. or systematic review/ or cochrane.jw. or (prisma or prospero).ti,ab,kf. or ((systemati* or scoping or umbrella or "structured literature") adj3 (review* or overview*)).ti,ab,kf. or (systemic* adj1 review*).ti,ab,kf. or ((systemati* or literature or database* or data-base*) adj10 search*).ti,ab,kf. or ((structured or comprehensive* or systemic*) adj3 search*).ti,ab,kf. or ((literature adj3 review*) and (search* or database* or data-base*)).ti,ab,kf. or (("data extraction" or "data source*") and "study selection").ti,ab,kf. or ("search strategy" and "selection criteria").ti,ab,kf. or ("data source*" and "data synthesis").ti,ab,kf. or (medline or pubmed or embase or cochrane).ab. or ((critical or rapid) adj2 (review* or overview* or synthes*)).ti. or (((critical* or rapid*) adj3 (review* or overview* or synthes*)) and (search* or database* or data-base*)).ab. or (metasynthes* or meta-synthes*).ti,ab,kf.

#6 - Randomized Controlled Trials

6. exp clinical trial/ or randomized controlled trial/ or exp clinical trials as topic/ or randomized controlled trials as topic/ or Random Allocation/ or Double-Blind Method/ or Single-Blind Method/ or (clinical trial, phase i or clinical trial, phase ii or clinical trial, phase iii or clinical trial, phase iv or controlled clinical trial or randomized controlled trial or multicenter study or clinical trial).pt. or random*.ti,ab. or (clinic* adj trial*).tw. or ((singl* or doubl* or treb* or tripl*) adj (blind$3 or mask$3)).tw. or Placebos/ or placebo*.tw.

#7 - Observational Studies (Cohort/Case-Control)

7. Epidemiologic studies/ or case control studies/ or exp cohort studies/ or Controlled Before-After Studies/ or Case control.tw. or cohort.tw. or Cohort analy$.tw. or (Follow up adj (study or studies)).tw. or (observational adj (study or studies)).tw. or Longitudinal.tw. or Retrospective*.tw. or prospective*.tw. or consecutive*.tw. or Cross sectional.tw. or Cross-sectional studies/ or historically controlled study/ or interrupted time series analysis/

#8 - Comparative Studies

8. Case-control Studies/ or clinical trial, phase ii/ or clinical trial, phase iii/ or clinical trial, phase iv/ or comparative study/ or control groups/ or controlled before-after studies/ or controlled clinical trial/ or double-blind method/ or historically controlled study/ or matched-pair analysis/ or single-blind method/ or (((control or controlled) adj6 (study or studies or trial)) or (compar* adj (study or studies)) or ((control or controlled) adj1 active) or "open label*" or ((double or two or three or multi or trial) adj (arm or arms)) or (allocat* adj10 (arm or arms)) or placebo* or "sham-control*" or ((single or double or triple or assessor) adj1 (blind* or masked)) or nonrandom* or "non-random*" or "quasi-experiment*" or "parallel group*" or "factorial trial" or "pretest posttest" or (phase adj5 (study or trial)) or (case* adj6 (matched or control*)) or (match* adj6 (pair or pairs or cohort* or control* or group* or healthy or age or sex or gender or patient* or subject* or participant*)) or (propensity adj6 (scor* or match*))).ti,ab,kf. or (confounding adj6 adjust*).ti,ab. or (versus or vs or compar*).ti. or ((exp cohort studies/ or epidemiologic studies/ or multicenter study/ or observational study/ or seroepidemiologic studies/ or (cohort* or "follow up" or followup or longitudinal* or prospective* or retrospective* or observational* or multicent* or "multi-cent*" or consecutive*).ti,ab,kf.) and ((group or groups or subgroup* or versus or vs or compar*).ti,ab,kf. or ("odds ratio*" or "relative odds" or "risk ratio*" or "relative risk*" or aor or arr or rrr).ab. or (("OR" or "RR") adj6 CI).ab.))

________________________________________

Final Combined Searches

SR = Systematic Reviews

9. 4 and 5

RCT = Randomized Controlled Trials

10. (4 and 6) not 5

Observational = Observational Studies

11. (4 and (7 or 8)) not (5 or 6)

________________________________________

Search Filters Applied

Date Filter: March 1, 2016 - June 6, 2025

Publication Types: Exclude Comments, Editorials, Letters

Study Population: Human studies only (exclude animal studies)

**EMBASE Search Strategy: Cervical Radiculopathy Physical Examination Tests**

Search Strategy Overview

Database: EMBASE (Ovid Interface)

Date Range: March 1, 2016 - June 5, 2025

Population: Human studies only

Individual Search Sets

#1 - Condition (Cervical Radiculopathy)

exp Radiculopathy/ or Brachial Plexus Neuropathies/ or Brachial Plexus Neuritis/ or cervicobrachial*.ti,ab,kf. or "cervico brachial*".ti,ab,kf. or "cervical brachial*".ti,ab,kf. or ((cervic* or brachial*) adj3 (neuralg* or compress* or radiculop* or avulsion* or radiculitis* or radiculitides* or syndrome* or myelopath* or spondylos* or osteophytos* or stenosis* or degenerat* or neuritis*)).ti,ab,kf. or ((radiculalgia or radiculitis or radiculitides or radiculopath* or polyradiculopath* or neuralgia or "herniated disc*" or hernia or (radicular adj3 (pain* or neuralgia* or symptom* or syndrom*)) or ("nerve root" adj3 (pain* or inflammation* or disorder* or compression* or avulsion* or impingement))).ti,ab,kf. and (exp Cervical Vertebrae/ or exp Neck/ or cervical.ti,ab,kf. or cervico*.ti,ab,kf. or neck.ti,ab,kf.)) or ((Spinal Cord Compression/ or Intervertebral Disc Displacement/ or Spinal Stenosis/ or Intervertebral Disc Degeneration/ or Spondylosis/) and (exp Neck/ or Neck Pain/ or Neck Injuries/ or exp Cervical Vertebrae/ or Cervical Plexus/ or exp Spinal Injuries/ or Cervical Cord/ or exp Spinal Cord Injuries/))

#2 - Physical Examination/Tests

Physical Examination/ OR Physical Therapy Specialty/ OR Physical Therapists/ OR exp Neurologic Examination/ OR physiotherap*.ti,ab,kf. OR (medical* adj3 examin*).ti,ab,kf. OR provocat*.ti,ab,kf. OR movement*.ti,ab,kf. OR abduction*.ti,ab,kf. OR spurling*.ti,ab,kf. OR "arm squeeze test*".ti,ab,kf. OR wainner.ti,ab,kf. OR (test adj3 cluster).ti,ab,kf. OR "neck tornado test*".ti,ab,kf. OR (tendon* adj3 reflex*).ti,ab,kf. OR manipulat*.ti,ab,kf. OR ((manual OR physical* OR neurodynamic OR "neuro dynamic" OR neurolog*) adj3 (measur* OR assess* OR rating* OR test* OR exam*)).ti,ab,kf. OR (((sensor* OR sensat*) adj3 (impair* OR dysfunction* OR abnormal*)).ti,ab,kf.) OR valsalva* maneuv*.ti,ab,kf. OR (((elvey OR davidson* OR distraction* OR relief* OR tension OR motor OR "upper limb" OR "brachial plexus") adj3 test*).ti,ab,kf.) OR ultt*.ti,ab,kf. OR ("shoulder abduction" adj3 (sign OR test*)).ti,ab,kf.

#3 - Diagnostic Accuracy Terms

exp "Sensitivity and Specificity"/ or (sensitivity or sensitive or specificity).ti,ab. or (ROC-curve or receiver-operator*).ti,ab. or (likelihood or LR*).ti,ab. or exp Diagnostic Errors/ or (inter-observer or intra-observer or interobserver or intraobserver or validity or kappa or reliability).ti,ab. or reproducibility.ti,ab. or (test adj2 (re-test or retest)).ti,ab. or "Reproducibility of Results"/ or accuracy.ti,ab. or Diagnosis, Differential/ or Validation Studies.pt. or exp "Predictive Value of Tests"/ or ppv.ti,ab,kf. or npv.ti,ab,kf. or ((false or true) adj3 (negative or positive)).ti,ab.

#4 - Combined Core Search

1 and 2 and 3

#5 - Date Limit

limit 4 to dd=20160301-20250604

#6 - Human Studies Only

limit 5 to human

#7 - Systematic Reviews

meta-analysis/ or meta-analysis as topic/ or (metaanaly* or meta-analy* or metanaly*).ti,ab,kf. or systematic review/ or cochrane.jw. or (prisma or prospero).ti,ab,kf. or ((systemati* or scoping or umbrella or "structured literature") adj3 (review* or overview*)).ti,ab,kf. or (systemic* adj1 review*).ti,ab,kf. or ((systemati* or literature or database* or data-base*) adj10 search*).ti,ab,kf. or ((structured or comprehensive* or systemic*) adj3 search*).ti,ab,kf. or ((literature adj3 review*) and (search* or database* or data-base*)).ti,ab,kf. or (("data extraction" or "data source*") and "study selection").ti,ab,kf. or ("search strategy" and "selection criteria").ti,ab,kf. or ("data source*" and "data synthesis").ti,ab,kf. or (medline or pubmed or embase or cochrane).ab. or ((critical or rapid) adj2 (review* or overview* or synthes*)).ti. or (((critical* or rapid*) adj3 (review* or overview* or synthes*)) and (search* or database* or data-base*)).ab. or (metasynthes* or meta-synthes*).ti,ab,kf.

#8 - Randomized Controlled Trials

exp clinical trial/ or randomized controlled trial/ or exp clinical trials as topic/ or randomized controlled trials as topic/ or Random Allocation/ or Double-Blind Method/ or Single-Blind Method/ or (clinical trial, phase i or clinical trial, phase ii or clinical trial, phase iii or clinical trial, phase iv or controlled clinical trial or randomized controlled trial or multicenter study or clinical trial).pt. or random*.ti,ab. or (clinic* adj trial*).tw. or ((singl* or doubl* or treb* or tripl*) adj (blind$3 or mask$3)).tw. or Placebos/ or placebo*.tw.

#9 - Observational Studies (Cohort/Case-Control)

Epidemiologic studies/ or case control studies/ or exp cohort studies/ or Controlled Before-After Studies/ or Case control.tw. or cohort.tw. or Cohort analy$.tw. or (Follow up adj (study or studies)).tw. or (observational adj (study or studies)).tw. or Longitudinal.tw. or Retrospective*.tw. or prospective*.tw. or consecutive*.tw. or Cross sectional.tw. or Cross-sectional studies/ or historically controlled study/ or interrupted time series analysis/

#10 - Comparative Studies

Case-control Studies/ or clinical trial, phase ii/ or clinical trial, phase iii/ or clinical trial, phase iv/ or comparative study/ or control groups/ or controlled before-after studies/ or controlled clinical trial/ or double-blind method/ or historically controlled study/ or matched-pair analysis/ or single-blind method/ or (((control or controlled) adj6 (study or studies or trial)) or (compar* adj (study or studies)) or ((control or controlled) adj1 active) or "open label*" or ((double or two or three or multi or trial) adj (arm or arms)) or (allocat* adj10 (arm or arms)) or placebo* or "sham-control*" or ((single or double or triple or assessor) adj1 (blind* or masked)) or nonrandom* or "non-random*" or "quasi-experiment*" or "parallel group*" or "factorial trial" or "pretest posttest" or (phase adj5 (study or trial)) or (case* adj6 (matched or control*)) or (match* adj6 (pair or pairs or cohort* or control* or group* or healthy or age or sex or gender or patient* or subject* or participant*)) or (propensity adj6 (scor* or match*))).ti,ab,kf. or (confounding adj6 adjust*).ti,ab. or (versus or vs or compar*).ti. or ((exp cohort studies/ or epidemiologic studies/ or multicenter study/ or observational study/ or seroepidemiologic studies/ or (cohort* or "follow up" or followup or longitudinal* or prospective* or retrospective* or observational* or multicent* or "multi-cent*" or consecutive*).ti,ab,kf.) and ((group or groups or subgroup* or versus or vs or compar*).ti,ab,kf. or ("odds ratio*" or "relative odds" or "risk ratio*" or "relative risk*" or aor or arr or rrr).ab. or (("OR" or "RR") adj6 CI).ab.))

Final Combined Searches

SR = Systematic Reviews

11 = 6 and 7

RCT = Randomized Controlled Trials

12 = (6 and 8) not 7

Observational = Observational Studies

13 = (6 and (9 or 10)) not (7 or 8)

Search Filters Applied

**CINAHL Search Strategy: Cervical Radiculopathy Physical Examination Tests**

Search Strategy Overview

Database: CINAHL Complete (EBSCOhost)

Date Range: March 2016 – June 5, 2025

________________________________________

Individual Search Sets

S1 - Condition (Cervical Radiculopathy)

S1 (MH "Radiculopathy") OR (MH "Brachial Plexus") OR (MH "Cervical Vertebrae") OR

TI ( cervicobrachial* OR "cervico brachial*" OR "cervical brachial*" ) OR

AB ( cervicobrachial* OR "cervico brachial*" OR "cervical brachial*" ) OR

TI ( (cervic* OR brachial*) N3 (neuralg* OR compress* OR radiculop* OR avulsion* OR radiculitis* OR syndrome* OR myelopath* OR spondylos* OR stenosis* OR degenerat* OR neuritis*) ) OR

AB ( (cervic* OR brachial*) N3 (neuralg* OR compress* OR radiculop* OR avulsion* OR radiculitis* OR syndrome* OR myelopath* OR spondylos* OR stenosis* OR degenerat* OR neuritis*) ) OR

( TI ( radiculalgia OR radiculitis OR radiculopath* OR polyradiculopath* OR neuralgia OR "herniated disc*" OR hernia ) OR

AB ( radiculalgia OR radiculitis OR radiculopath* OR polyradiculopath* OR neuralgia OR "herniated disc*" OR hernia ) OR

TI ( radicular N3 (pain* OR neuralg* OR symptom* OR syndrom*) ) OR

AB ( radicular N3 (pain* OR neuralg* OR symptom* OR syndrom*) ) OR

TI ( "nerve root" N3 (pain* OR inflammation* OR disorder* OR compression* OR avulsion* OR impingement) ) OR

AB ( "nerve root" N3 (pain* OR inflammation* OR disorder* OR compression* OR avulsion* OR impingement) ) ) AND

( TI ( cervical OR cervico* OR neck ) OR AB ( cervical OR cervico* OR neck ) ) OR

( (MH "Spinal Cord Compression") OR (MH "Intervertebral Disk") OR (MH "Spinal Stenosis") OR (MH "Spondylosis") OR

TI ( "spinal cord compression" OR "intervertebral disc" OR "spinal stenosis" OR "disc degeneration" OR spondylosis ) OR

AB ( "spinal cord compression" OR "intervertebral disc" OR "spinal stenosis" OR "disc degeneration" OR spondylosis ) ) AND

( (MH "Neck") OR (MH "Neck Pain") OR (MH "Cervical Vertebrae") OR

TI ( neck OR cervical OR "cervical spine" ) OR AB ( neck OR cervical OR "cervical spine" ) )

S2 - Physical Examination/Tests

S2 (MH "Physical Examination") OR (MH "Physical Therapy") OR (MH "Physical Therapists") OR

(MH "Neurologic Examination") OR (MH "Diagnostic Tests, Routine") OR

TI ( physiotherap* ) OR AB ( physiotherap* ) OR

TI ( medical* N3 examin* ) OR AB ( medical* N3 examin* ) OR

TI ( provocat* OR movement* OR abduction* OR spurling* ) OR

AB ( provocat* OR movement* OR abduction* OR spurling* ) OR

TI ( "arm squeeze test*" OR wainner OR "neck tornado test*" ) OR

AB ( "arm squeeze test*" OR wainner OR "neck tornado test*" ) OR

TI ( test N3 cluster ) OR AB ( test N3 cluster ) OR

TI ( tendon* N3 reflex* ) OR AB ( tendon* N3 reflex* ) OR

TI ( manipulat* ) OR AB ( manipulat* ) OR

TI ( (manual OR physical* OR neurodynamic OR "neuro dynamic" OR neurolog*) N3 (measur* OR assess* OR rating* OR test* OR exam*) ) OR

AB ( (manual OR physical* OR neurodynamic OR "neuro dynamic" OR neurolog*) N3 (measur* OR assess* OR rating* OR test* OR exam*) ) OR

TI ( (sensor* OR sensat*) N3 (impair* OR dysfunction* OR abnormal*) ) OR

AB ( (sensor* OR sensat*) N3 (impair* OR dysfunction* OR abnormal*) ) OR

TI ( "valsalva* maneuv*" ) OR AB ( "valsalva* maneuv*" ) OR

TI ( (elvey OR davidson* OR distraction* OR relief* OR tension OR motor OR "upper limb" OR "brachial plexus") N3 test* ) OR

AB ( (elvey OR davidson* OR distraction* OR relief* OR tension OR motor OR "upper limb" OR "brachial plexus") N3 test* ) OR

TI ( ultt* ) OR AB ( ultt* ) OR

TI ( "shoulder abduction" N3 (sign OR test*) ) OR AB ( "shoulder abduction" N3 (sign OR test*) )

S3 - Diagnostic Accuracy Terms

S3 (MH "Sensitivity and Specificity") OR (MH "Diagnostic Errors") OR

(MH "Reproducibility of Results") OR (MH "Predictive Value of Tests") OR

TI ( sensitivity OR sensitive OR specificity ) OR AB ( sensitivity OR sensitive OR specificity ) OR

TI ( "ROC curve" OR "receiver operat*" ) OR AB ( "ROC curve" OR "receiver operat*" ) OR

TI ( likelihood ) OR AB ( likelihood ) OR

TI ( "inter observer" OR "intra observer" OR interobserver OR intraobserver ) OR

AB ( "inter observer" OR "intra observer" OR interobserver OR intraobserver ) OR

TI ( validity OR kappa OR reliability OR reproducibility ) OR

AB ( validity OR kappa OR reliability OR reproducibility ) OR

TI ( test N2 ("re-test" OR retest) ) OR AB ( test N2 ("re-test" OR retest) ) OR

TI ( accuracy ) OR AB ( accuracy ) OR

TI ( "differential diagnosis" OR "validation stud*" OR "predictive value*" ) OR

AB ( "differential diagnosis" OR "validation stud*" OR "predictive value*" ) OR

TI ( ppv OR npv ) OR AB ( ppv OR npv ) OR

TI ( (false OR true) N3 (negative OR positive) ) OR AB ( (false OR true) N3 (negative OR positive) )

S4 - Combined Core Search

S4 S1 AND S2 AND S3

S5 - Systematic Reviews

S5 (MH "Meta Analysis") OR (MH "Systematic Review") OR (PT "systematic review") OR

TI ( metaanaly* OR "meta analy*" OR metanaly* ) OR AB ( metaanaly* OR "meta analy*" OR metanaly* ) OR

TI ( "systematic review*" ) OR AB ( "systematic review*" ) OR

TI ( (systemati* OR scoping OR umbrella OR "structured literature") N3 (review* OR overview*) ) OR

AB ( (systemati* OR scoping OR umbrella OR "structured literature") N3 (review* OR overview*) ) OR

TI ( systemic* N1 review* ) OR AB ( systemic* N1 review* ) OR

TI ( (systemati* OR literature OR database*) N10 search* ) OR

AB ( (systemati* OR literature OR database*) N10 search* ) OR

TI ( (structured OR comprehensive* OR systemic*) N3 search* ) OR

AB ( (structured OR comprehensive* OR systemic*) N3 search* ) OR

TI ( literature N3 review* ) AND ( TI ( search* OR database* ) OR AB ( search* OR database* ) ) OR

AB ( literature N3 review* ) AND ( TI ( search* OR database* ) OR AB ( search* OR database* ) ) OR

TI ( ("data extraction" OR "data source*") AND "study selection" ) OR

AB ( ("data extraction" OR "data source*") AND "study selection" ) OR

TI ( "search strategy" AND "selection criteria" ) OR AB ( "search strategy" AND "selection criteria" ) OR

TI ( "data source*" AND "data synthesis" ) OR AB ( "data source*" AND "data synthesis" ) OR

AB ( medline OR pubmed OR embase OR cochrane ) OR

TI ( (critical OR rapid) N2 (review* OR overview* OR synthes*) ) OR

AB ( (critical OR rapid) N2 (review* OR overview* OR synthes*) ) OR

TI ( metasynthes* OR "meta synthes*" ) OR AB ( metasynthes* OR "meta synthes*" ) OR

TI ( prisma OR prospero ) OR AB ( prisma OR prospero )

S6 - Randomized Controlled Trials

S6 (MH "Clinical Trials+") OR (MH "Random Assignment") OR (MH "Random Sample+") OR

(MH "Crossover Design") OR (MH "Clinical Trial Registry") OR (PT "clinical trial") OR

(PT "randomized controlled trial") OR

TI ( random* ) OR AB ( random* ) OR

TI ( "clinical trial*" ) OR AB ( "clinical trial*" ) OR

TI ( trial ) OR

TI ( (singl* OR doubl* OR tripl*) N1 (blind* OR mask*) ) OR

AB ( (singl* OR doubl* OR tripl*) N1 (blind* OR mask*) ) OR

TI ( placebo* ) OR AB ( placebo* ) OR

TI ( "controlled trial*" OR "randomized controlled trial*" OR RCT ) OR

AB ( "controlled trial*" OR "randomized controlled trial*" OR RCT ) OR

TI ( allocation OR randomization ) OR AB ( allocation OR randomization ) OR

TI ( (control* OR controlled) N6 (trial OR study) ) OR

AB ( (control* OR controlled) N6 (trial OR study) ) OR

TI ( crossover OR "cross over" ) OR AB ( crossover OR "cross over" )

S7 - Observational Studies

S7 (MH "Prospective Studies+") OR (MH "Retrospective Studies+") OR (MH "Case Control Studies+") OR

(MH "Longitudinal Studies") OR (MH "Cohort Studies") OR (MH "Cross Sectional Studies") OR

(MH "Comparative Studies") OR (MH "Epidemiologic Studies+") OR

TI ( "case control*" OR "case-control*" ) OR AB ( "case control*" OR "case-control*" ) OR

TI ( cohort* ) OR AB ( cohort* ) OR

TI ( "follow up" OR followup ) OR AB ( "follow up" OR followup ) OR

TI ( longitudinal* OR prospective* OR retrospective* ) OR

AB ( longitudinal* OR prospective* OR retrospective* ) OR

TI ( observational* OR "cross sectional*" OR "cross-sectional*" ) OR

AB ( observational* OR "cross sectional*" OR "cross-sectional*" ) OR

TI ( multicent* OR "multi-cent*" OR consecutive* ) OR

AB ( multicent* OR "multi-cent*" OR consecutive* ) OR

TI ( "epidemiologic* stud*" OR "cohort stud*" OR "observational stud*" ) OR

AB ( "epidemiologic* stud*" OR "cohort stud*" OR "observational stud*" )

S8 - Comparative Studies

S8 TI ( "control* stud*" OR "comparative stud*" ) OR AB ( "control* stud*" OR "comparative stud*" ) OR

TI ( (control OR controlled) N1 active ) OR AB ( (control OR controlled) N1 active ) OR

TI ( "open label*" ) OR AB ( "open label*" ) OR

TI ( (double OR two OR three OR multi OR trial) N1 (arm OR arms) ) OR

AB ( (double OR two OR three OR multi OR trial) N1 (arm OR arms) ) OR

TI ( allocat* N10 (arm OR arms) ) OR AB ( allocat* N10 (arm OR arms) ) OR

TI ( "sham control*" ) OR AB ( "sham control*" ) OR

TI ( "parallel group*" OR "factorial trial" ) OR AB ( "parallel group*" OR "factorial trial" ) OR

TI ( phase N5 (study OR trial) ) OR AB ( phase N5 (study OR trial) ) OR

TI ( case* N6 (matched OR control*) ) OR AB ( case* N6 (matched OR control*) ) OR

TI ( match* N6 (pair OR pairs OR cohort* OR control* OR group* OR patient* OR subject*) ) OR

AB ( match* N6 (pair OR pairs OR cohort* OR control* OR group* OR patient* OR subject*) ) OR

TI ( propensity N6 (scor* OR match*) ) OR AB ( propensity N6 (scor* OR match*) ) OR

TI ( versus OR vs OR compar* ) OR

AB ( "odds ratio*" OR "relative odds" OR "risk ratio*" OR "relative risk*" )

________________________________________

Final Combined Searches

SR = Systematic Reviews

S9 S4 AND S5

RCT = Randomized Controlled Trials

S10 (S4 AND S6) NOT S5

Observational = Observational Studies

S11 (S4 AND (S7 OR S8)) NOT (S5 OR S6)

________________________________________

Limiters and Filters

Date Limitation

March 2016 – June 2025

Language Filter

Language: English

Subject Population

Human (exclude animal studies if available in interface)

**PubMed Search Strategy: Cervical Radiculopathy Physical Examination Tests**

Search Strategy Overview

Database: PubMed (NCBI Interface)

Date Range: March 1, 2016 - June 5, 2025

Document Types: Exclude Comments, Editorials, Letters

Language: English

Population: Human studies only

________________________________________

Individual Search Sets

#1 - Condition (Cervical Radiculopathy)

#1 ("Radiculopathy"[Mesh] OR "Brachial Plexus Neuropathies"[Mesh] OR "Brachial Plexus Neuritis"[Mesh] OR cervicobrachial*[tiab] OR "cervico brachial*"[tiab] OR "cervical brachial*"[tiab] OR ((cervic*[tiab] OR brachial*[tiab]) AND (neuralg*[tiab] OR compress*[tiab] OR radiculop*[tiab] OR avulsion*[tiab] OR radiculitis*[tiab] OR radiculitides*[tiab] OR syndrome*[tiab] OR myelopath*[tiab] OR spondylos*[tiab] OR osteophytos*[tiab] OR stenosis*[tiab] OR degenerat*[tiab] OR neuritis*[tiab])) OR ((radiculalgia[tiab] OR radiculitis[tiab] OR radiculitides[tiab] OR radiculopath*[tiab] OR polyradiculopath*[tiab] OR neuralgia[tiab] OR "herniated disc*"[tiab] OR hernia[tiab] OR (radicular[tiab] AND (pain*[tiab] OR neuralgia*[tiab] OR symptom*[tiab] OR syndrom*[tiab])) OR ("nerve root"[tiab] AND (pain*[tiab] OR inflammation*[tiab] OR disorder*[tiab] OR compression*[tiab] OR avulsion*[tiab] OR impingement[tiab]))) AND ("Cervical Vertebrae"[Mesh] OR "Neck"[Mesh] OR cervical[tiab] OR cervico*[tiab] OR neck[tiab])) OR (("Spinal Cord Compression"[Mesh] OR "Intervertebral Disc Displacement"[Mesh] OR "Spinal Stenosis"[Mesh] OR "Intervertebral Disc Degeneration"[Mesh] OR "Spondylosis"[Mesh]) AND ("Neck"[Mesh] OR "Neck Pain"[Mesh] OR "Neck Injuries"[Mesh] OR "Cervical Vertebrae"[Mesh] OR "Brachial Plexus"[Mesh] OR "Spinal Injuries"[Mesh] OR "Spinal Cord Injuries"[Mesh])))

#2 - Physical Examination/Tests

#2 ("Physical Examination"[Mesh] OR "Physical Therapy Specialty"[Mesh] OR "Physical Therapists"[Mesh] OR "Neurologic Examination"[Mesh] OR physiotherap*[tiab] OR (medical*[tiab] AND examin*[tiab]) OR provocat*[tiab] OR movement*[tiab] OR abduction*[tiab] OR spurling*[tiab] OR "arm squeeze test*"[tiab] OR wainner[tiab] OR (test[tiab] AND cluster[tiab]) OR "neck tornado test*"[tiab] OR (tendon*[tiab] AND reflex*[tiab]) OR manipulat*[tiab] OR ((manual[tiab] OR physical*[tiab] OR neurodynamic[tiab] OR "neuro dynamic"[tiab] OR neurolog*[tiab]) AND (measur*[tiab] OR assess*[tiab] OR rating*[tiab] OR test*[tiab] OR exam*[tiab])) OR ((sensor*[tiab] OR sensat*[tiab]) AND (impair*[tiab] OR dysfunction*[tiab] OR abnormal*[tiab])) OR "valsalva* maneuv*"[tiab] OR ((elvey[tiab] OR davidson*[tiab] OR distraction*[tiab] OR relief*[tiab] OR tension[tiab] OR motor[tiab] OR "upper limb"[tiab] OR "brachial plexus"[tiab]) AND test*[tiab]) OR ultt*[tiab] OR ("shoulder abduction"[tiab] AND (sign[tiab] OR test*[tiab])))

#3 - Diagnostic Accuracy Terms

#3 ("Sensitivity and Specificity"[Mesh] OR sensitivity[tiab] OR sensitive[tiab] OR specificity[tiab] OR "ROC curve"[tiab] OR "receiver operator*"[tiab] OR "receiver operat*"[tiab] OR likelihood[tiab] OR "Diagnostic Errors"[Mesh] OR "inter observer"[tiab] OR "intra observer"[tiab] OR interobserver[tiab] OR intraobserver[tiab] OR validity[tiab] OR kappa[tiab] OR reliability[tiab] OR reproducibility[tiab] OR (test[tiab] AND ("re-test"[tiab] OR retest[tiab])) OR "Reproducibility of Results"[Mesh] OR accuracy[tiab] OR "Diagnosis, Differential"[Mesh] OR "Predictive Value of Tests"[Mesh] OR ppv[tiab] OR npv[tiab] OR ((false[tiab] OR true[tiab]) AND (negative[tiab] OR positive[tiab])))

#4 - Combined Core Search with Filters

#4 (#1 AND #2 AND #3) AND ("2016/03/01"[PDAT] : "2025/06/06"[PDAT]) NOT ("Comment"[PT] OR "Editorial"[PT] OR "Letter"[PT]) AND "humans"[MeSH Terms] AND English[lang]

#5 - Systematic Reviews

#5 ("Meta-Analysis"[PT] OR "Meta-Analysis as Topic"[Mesh] OR "systematic review"[PT] OR metaanaly*[tiab] OR "meta analy*"[tiab] OR metanaly*[tiab] OR "systematic review*"[tiab] OR prisma[tiab] OR prospero[tiab] OR ((systemati*[tiab] OR scoping[tiab] OR umbrella[tiab] OR "structured literature"[tiab]) AND (review*[tiab] OR overview*[tiab])) OR (systemic*[tiab] AND review*[tiab]) OR ((systemati*[tiab] OR literature[tiab] OR database*[tiab] OR "data base*"[tiab]) AND search*[tiab]) OR ((structured[tiab] OR comprehensive*[tiab] OR systemic*[tiab]) AND search*[tiab]) OR ((literature[tiab] AND review*[tiab]) AND (search*[tiab] OR database*[tiab] OR "data base*"[tiab])) OR (("data extraction"[tiab] OR "data source*"[tiab]) AND "study selection"[tiab]) OR ("search strategy"[tiab] AND "selection criteria"[tiab]) OR ("data source*"[tiab] AND "data synthesis"[tiab]) OR medline[tiab] OR pubmed[tiab] OR embase[tiab] OR ((critical[tiab] OR rapid[tiab]) AND (review*[tiab] OR overview*[tiab] OR synthes*[tiab])) OR metasynthes*[tiab] OR "meta synthes*"[tiab])

#6 - Randomized Controlled Trials

#6 ("Clinical Trial"[PT] OR "Randomized Controlled Trial"[PT] OR "Clinical Trials as Topic"[Mesh] OR "Random Allocation"[Mesh] OR "Double-Blind Method"[Mesh] OR "Single-Blind Method"[Mesh] OR random*[tiab] OR "clinical trial*"[tiab] OR trial[ti] OR ((singl*[tiab] OR doubl*[tiab] OR tripl*[tiab]) AND (blind*[tiab] OR mask*[tiab])) OR "Placebos"[Mesh] OR placebo*[tiab] OR "controlled trial*"[tiab] OR "randomized controlled trial*"[tiab] OR RCT[tiab] OR allocation[tiab] OR randomization[tiab] OR "control group*"[tiab] OR ((control*[tiab] OR controlled[tiab]) AND (trial[tiab] OR study[tiab])) OR "comparative study"[tiab] OR crossover[tiab] OR "cross over"[tiab] OR "parallel group*"[tiab] OR "factorial trial"[tiab] OR versus[ti] OR vs[ti])

#7 - Observational Studies (Cohort/Case-Control)

#7 ("Epidemiologic Studies"[Mesh] OR "Case-Control Studies"[Mesh] OR "Cohort Studies"[Mesh] OR "Cross-Sectional Studies"[Mesh] OR "case control*"[tiab] OR "case-control*"[tiab] OR cohort*[tiab] OR "follow up"[tiab] OR followup[tiab] OR longitudinal*[tiab] OR prospective*[tiab] OR retrospective*[tiab] OR observational*[tiab] OR "cross sectional*"[tiab] OR "cross-sectional*"[tiab] OR multicent*[tiab] OR "multi-cent*"[tiab] OR consecutive*[tiab] OR "epidemiologic* stud*"[tiab] OR "cohort stud*"[tiab] OR "observational stud*"[tiab])

#8 - Comparative Studies

#8 ("Case-Control Studies"[Mesh] OR "Comparative Study"[PT] OR "controlled study"[tiab] OR "comparative study"[tiab] OR "controlled trial*"[tiab] OR ((control[tiab] OR controlled[tiab]) AND (study[tiab] OR studies[tiab] OR trial[tiab])) OR ((control[tiab] OR controlled[tiab]) AND active[tiab]) OR "open label*"[tiab] OR ((double[tiab] OR two[tiab] OR three[tiab] OR multi[tiab] OR trial[tiab]) AND (arm[tiab] OR arms[tiab])) OR (allocat*[tiab] AND (arm[tiab] OR arms[tiab])) OR placebo*[tiab] OR "sham control*"[tiab] OR ((single[tiab] OR double[tiab] OR triple[tiab] OR assessor[tiab]) AND (blind*[tiab] OR masked[tiab])) OR nonrandom*[tiab] OR "non-random*"[tiab] OR "quasi-experiment*"[tiab] OR crossover[tiab] OR "cross over"[tiab] OR "parallel group*"[tiab] OR "factorial trial"[tiab] OR (phase[tiab] AND (study[tiab] OR trial[tiab])) OR (case*[tiab] AND (matched[tiab] OR control*[tiab])) OR (match*[tiab] AND (pair[tiab] OR pairs[tiab] OR cohort*[tiab] OR control*[tiab] OR group*[tiab] OR healthy[tiab] OR age[tiab] OR sex[tiab] OR gender[tiab] OR patient*[tiab] OR subject*[tiab] OR participant*[tiab])) OR (propensity[tiab] AND (scor*[tiab] OR match*[tiab])) OR versus[ti] OR vs[ti] OR compar*[ti] OR (compar*[tiab] AND study[tiab]) OR ((cohort*[tiab] OR "follow up"[tiab] OR followup[tiab] OR longitudinal*[tiab] OR prospective*[tiab] OR retrospective*[tiab] OR observational*[tiab] OR multicent*[tiab] OR "multi-cent*"[tiab] OR consecutive*[tiab]) AND (group[tiab] OR groups[tiab] OR subgroup*[tiab] OR versus[tiab] OR vs[tiab] OR compar*[tiab] OR "odds ratio*"[tiab] OR "relative odds"[tiab] OR "risk ratio*"[tiab] OR "relative risk*"[tiab] OR "rate ratio"[tiab] OR aor[tiab] OR arr[tiab] OR rrr[tiab])))

________________________________________

Final Combined Searches

SR = Systematic Reviews

#9 #4 AND #5

RCT = Randomized Controlled Trials

#10 (#4 AND #6) NOT #5

Observational = Observational Studies

#11 (#4 AND (#7 OR #8)) NOT (#5 OR #6)

________________________________________

Search Filters Applied

Date Filter: March 1, 2016 - June 6, 2025

Publication Types: Exclude Comments, Editorials, Letters

Study Population: Human studies only

Language: English

Database Interface: PubMed (NCBI)

**Web of Science Search Strategy: Cervical Radiculopathy Physical Examination Tests**

Search Strategy Overview

Database: Web of Science Core Collection

Date Range: March 1, 2016 - June 5, 2025

Document Types: Articles, Reviews (excluding Meeting Abstracts, Editorial Material, Letters)

________________________________________

Individual Search Sets

#1 - Condition (Cervical Radiculopathy)

TS=(cervicobrachial* OR "cervico brachial*" OR "cervical brachial*" OR

((cervic* OR brachial*) NEAR/3 (neuralg* OR compress* OR radiculop* OR avulsion* OR radiculitis* OR syndrome* OR myelopath* OR spondylos* OR stenosis* OR degenerat* OR neuritis*)) OR

((radiculalgia OR radiculitis OR radiculopath* OR polyradiculopath* OR neuralgia OR "herniated disc*" OR hernia OR

(radicular NEAR/3 (pain* OR neuralg* OR symptom* OR syndrom*)) OR

("nerve root" NEAR/3 (pain* OR inflammation* OR disorder* OR compression* OR avulsion* OR impingement))) AND

(cervical OR cervico* OR neck)) OR

(("spinal cord compression" OR "intervertebral disc displacement" OR "spinal stenosis" OR "disc degeneration" OR spondylosis) AND (neck OR cervical OR "cervical spine")))

#2 - Physical Examination/Tests

TS=(physiotherap* OR

(medical* NEAR/3 examin*) OR provocat* OR movement* OR abduction* OR spurling* OR

"arm squeeze test*" OR wainner OR (test NEAR/3 cluster) OR "neck tornado test*" OR

(tendon* NEAR/3 reflex*) OR manipulat* OR

((manual OR physical* OR neurodynamic OR "neuro dynamic" OR neurolog*) NEAR/3 (measur* OR assess* OR rating* OR test* OR exam*)) OR

((sensor* OR sensat*) NEAR/3 (impair* OR dysfunction* OR abnormal*)) OR

"valsalva* maneuv*" OR

((elvey OR davidson* OR distraction* OR relief* OR tension OR motor OR "upper limb" OR "brachial plexus") NEAR/3 test*) OR

ultt* OR ("shoulder abduction" NEAR/3 (sign OR test*)))

#3 - Diagnostic Accuracy Terms

TS=(sensitivity OR sensitive OR specificity OR "ROC curve" OR "receiver operat*" OR likelihood OR

"inter observer" OR "intra observer" OR interobserver OR intraobserver OR validity OR kappa OR reliability OR reproducibility OR

(test NEAR/2 ("re-test" OR retest)) OR accuracy OR

"differential diagnosis" OR "validation stud*" OR "predictive value*" OR ppv OR npv OR

((false OR true) NEAR/3 (negative OR positive)))

#4 - Combined Core Search with Filters

#1 AND #2 AND #3

Refined by:

• Publication Years: 2016-2025

• Document Types: Article OR Review

• Language: English

• Exclude: Meeting Abstract OR Editorial Material OR Letter

#5 - Systematic Reviews

TS=(metaanaly* OR "meta analy*" OR metanaly* OR "systematic review*" OR

((systemati* OR scoping OR umbrella OR "structured literature") NEAR/3 (review* OR overview*)) OR

(systemic* NEAR/1 review*) OR

((systemati* OR literature OR database*) NEAR/10 search*) OR

((structured OR comprehensive* OR systemic*) NEAR/3 search*) OR

((literature NEAR/3 review*) AND (search* OR database*)) OR

(("data extraction" OR "data source*") AND "study selection") OR

("search strategy" AND "selection criteria") OR

("data source*" AND "data synthesis") OR

(medline OR pubmed OR embase OR cochrane) OR

((critical OR rapid) NEAR/2 (review* OR overview* OR synthes*)) OR

(metasynthes* OR "meta synthes*") OR prisma OR prospero)

#6 - Randomized Controlled Trials

TS=(random* OR "clinical trial*" OR

((singl* OR doubl* OR tripl*) NEAR/1 (blind* OR mask*)) OR placebo* OR

"controlled trial*" OR "randomized controlled trial*" OR RCT OR

"double blind*" OR "single blind*" OR

((control* OR controlled) NEAR/6 (trial OR study)) OR

"phase II" OR "phase III" OR "phase IV" OR

allocation OR randomization)

#7 - Observational Studies (Cohort/Case-Control)

TS=("case control*" OR cohort* OR "follow up" OR followup OR longitudinal* OR

prospective* OR retrospective* OR observational* OR "cross sectional*" OR

multicent* OR "multi-cent*" OR consecutive* OR

"epidemiologic* stud*" OR "cohort stud*" OR "case control stud*" OR

"observational stud*" OR "longitudinal stud*")

#8 - Comparative Studies

TS=(("control* stud*" OR "comparative stud*" OR "controlled trial*") OR

((control OR controlled) NEAR/1 active) OR "open label*" OR

((double OR two OR three OR multi OR trial) NEAR/1 (arm OR arms)) OR

(allocat* NEAR/10 (arm OR arms)) OR placebo* OR "sham control*" OR

((single OR double OR triple OR assessor) NEAR/1 (blind* OR mask*)) OR

nonrandom* OR "non-random*" OR "quasi-experiment*" OR crossover OR "cross over" OR

"parallel group*" OR "factorial trial" OR (phase NEAR/5 (study OR trial)) OR

(case* NEAR/6 (matched OR control*)) OR

(match* NEAR/6 (pair OR pairs OR cohort* OR control* OR group* OR healthy OR age OR sex OR gender OR patient* OR subject* OR participant*)) OR

(propensity NEAR/6 (scor* OR match*)) OR versus OR vs OR compar* OR

(("odds ratio*" OR "relative odds" OR "risk ratio*" OR "relative risk*" OR "rate ratio" OR aor OR arr OR rrr) AND (group OR groups OR subgroup* OR versus OR vs OR compar*)))

________________________________________

Final Combined Searches

SR = Systematic Reviews

#4 AND #5

RCT = Randomized Controlled Trials

(#4 AND #6) NOT #5

Observational = Observational Studies

(#4 AND (#7 OR #8)) NOT (#5 OR #6)
