# Supplementary material for: Diagnostic accuracy of physical examination tests for painful cervical radiculopathy: update of a systematic review and meta-analysis
Source: BMC Musculoskelet Disord. 2026 Feb 13;27:338. doi: 10.1186/s12891-026-09551-0 (PMC13088722; doi:10.1186/s12891-026-09551-0)
Supplement: Supplementary file 2 — Supplementary Material 2. [file 12891_2026_9551_MOESM2_ESM.docx]

**APPENDIX 2: QUADAS-2 signalling questions and guidance for assessors**

**Phase 1: please state the review question:**

| *Patients (setting, intended use of index test, presentation, prior testing):*  Patients with radicular arm and neck pain in primary or secondary care |
| --- |
| *Index test(s):*  specific tests carried out during the physical examination for the diagnosis of CR: i.e.:  Spurling, Valsalva, ULNT, Shoulder abduction relief, traction, reflex, key muscles |
| *Reference standard and target condition:*  (Physical examination combined with) MRI / CT and or surgery |

**Phase 2: Draw a flow diagram for the primary study**

|  |
| --- |

**Phase 3: Risk of bias and applicability judgments**

*QUADAS-2 is structured so that 4 key domains are each rated in terms of the risk of bias and the concern regarding applicability to the research question (as defined above). Each key domain has a set of signalling questions to help reach the judgments regarding bias and applicability.*

| **DOMAIN 1: PATIENT SELECTION**  **A. Risk of Bias**   \| Describe methods of patient selection:  *Please describe the method as you understand it from the description in the manuscript.* \| \| --- \|   *From the answers to the signaling questions below, please derive a final score. The lowest score should determine the final score*   - Was a consecutive or random sample of patients enrolled? Yes/No/Unclear - Was a case-control design avoided? Yes/No/Unclear - Did the study avoid inappropriate exclusions? Yes/No/Unclear   **Could the selection of patients have introduced bias? RISK: LOW / HIGH / UNCLEAR**  **B. Concerns regarding applicability**   \| Describe included patients (prior testing, presentation, intended use of index test and setting)**:**  *Please describe the included patients as you understand them from the description in the manuscript.* \| \| --- \|   **Is there concern that the included patients do not match the review question?**  *Do you feel the included patients might have disorders not related to the review question? Eg. if the objective is to differentiate between NonSpecificArmPain and CR that is okay. But not so if the included patients might have completely unrelated disorders or have a spectrum of the disorder too different from the review question*  **CONCERN: LOW / HIGH / UNCLEAR** |
| --- | --- | --- |

| **DOMAIN 2: INDEX TEST(S)**  **If more than one index test was used, please complete for each test**   \| 1. **Risk of Bias** \| \| --- \|  \| Describe the index test and how it was conducted and interpreted:  *Please describe the index test(s) and the manner of applying them as you understand it from the description in the manuscript* \| \| --- \|   *From the answers to the signaling questions below, please derive a final score. The lowest score should determine the final score*   - Were the index test results interpreted without knowledge of the results of the reference standard? Yes /No /Unclear - If a threshold was used, was it pre-specified? Yes /No /Unclear   **Could the conduct or interpretation of the index test have introduced bias?**  **RISK: LOW / HIGH / UNCLEAR**   1. **Concerns regarding applicability**   Is there concern that the index test, its conduct or interpretation differ from the review question?  *Do you feel the index test or its manner of applying or interpreting the outcome (pos/ neg scoring) is too different so the review question cannot be answered from the result?*  **CONCERN: LOW /HIGH/UNCLEAR** |
| --- | --- | --- |

| **DOMAIN 3: REFERENCE STANDARD**   1. **Risk of Bias**  \| Describe the reference standard and how it was conducted and interpreted:  Please describe the reference standard(s) and the manner of applying and interpreting the outcome (pos/neg) as you understand it from the description in the manuscript.  In the absence of a true gold standard we state that the combination of a neurological examination (consisting of testing of tendon reflexes, manual muscle testing of key muscles for muscle weakness or atrophy and testing for sensory deficits) and results from MRI/ CT imagingand/or the postoperative results is to be seen as correctly classifying the target condition. A sole assessment of an MRI / CT (eg. by a radiologist) potentially has too many false positives and is therefore usually to be scored as “Unclear” \| \| --- \|   *From the answers to the signaling questions below, please derive a final score. The lowest score should determine the final score*   - Is the reference standard likely to correctly classify the target condition?   Yes /No /Unclear   - Where the reference standard results interpreted without knowledge of the results of the index test?   Yes /No /Unclear  **Could the reference standard, its conduct, or its interpretation have introduced bias?**  **RISK: LOW / HIGH / UNCLEAR**   1. **Concerns regarding applicability**   Is there concern that the target condition as defined by the reference standard does not match the review question?  *Do you feel the reference test itself or its manner of applying or interpreting the outcome (pos/ neg scoring) is too different so the review question cannot be answered from the result?*  **CONCERN: LOW / HIGH / UNCLEAR** |
| --- | --- |

| **DOMAIN 4: FLOW AND TIMING**   1. **Risk of Bias**  \| Describe any patients who did not receive the index test(s) and/or reference standard or who were excluded from the 2x2 table (refer to flow diagram):  Describe the time interval and any interventions between index test(s) and reference standard: \| \| --- \|   *From the answers to the signaling questions below, please derive a final score. The lowest score should determine the final score*   \| - Was there an appropriate interval **(< 1week)** between index test(s) and reference standard? - Did all patients receive a reference standard? - Did patients receive the same reference standard? - Were all patients included in the analysis? \| Yes /No /Unclear  Yes /No /Unclear  Yes /No /Unclear  Yes /No /Unclear \| \| --- \| --- \|   **Could the patient flow have introduced bias?** **RISK: LOW /HIGH/UNCLEAR** |
| --- | --- | --- | --- |
