# Supplementary material for: Diagnostic accuracy of physical examination tests for painful cervical radiculopathy: update of a systematic review and meta-analysis
Source: BMC Musculoskelet Disord. 2026 Feb 13;27:338. doi: 10.1186/s12891-026-09551-0 (PMC13088722; doi:10.1186/s12891-026-09551-0)
Supplement: Supplementary file 4 — Supplementary Material 4. [file 12891_2026_9551_MOESM4_ESM.docx]

Appendix 3. GRADE certainty of the evidence tables.

Summary of findings for: Spurling’s test

| № of studies (№ of patients) | Study design | Factors that may decrease certainty of evidence | | | | | Test accuracy CoE |
| --- | --- | --- | --- | --- | --- | --- | --- |
|  |  | Risk of bias | Indirectness | Inconsistency | Imprecision | Publication bias |  |
| 5 studies 308 patients | Combination of case-control, prospective and retrospective-type accuracy study | serious^a^ | not serious | very serious^b,c^ | serious^d^ | none | ⨁◯◯◯ Very low^a,b,c,d^ |
|  |  |  |  |  |  |  |  |
|  |  |  |  |  |  |  |  |

#### Explanations

a. >1 study with "unclear" or "high" risk of bias

b. Conflicting accuracy measures

c. wide 95% confidence intervals

d. Low number of included patients

Summary of findings for: ULNT1

| № of studies (№ of patients) | Study design | Factors that may decrease certainty of evidence | | | | | Test accuracy CoE |
| --- | --- | --- | --- | --- | --- | --- | --- |
|  |  | Risk of bias | Indirectness | Inconsistency | Imprecision | Publication bias |  |
| 3 studies 266 patients | Combination of case-control and prospective type accuracy study | serious^a^ | not serious | very serious^b,c^ | serious^d^ | none | ⨁◯◯◯ Very low^a,b,c,d^ |
|  |  |  |  |  |  |  |  |
|  |  |  |  |  |  |  |  |

#### Explanations

a. 1 study with high risk of bias

b. Conflicting accuracy measures

c. wide 95% confidence intervals

d. low number of included patients

| Summary of findings for: 4 combined ULNTs № of studies (№ of patients) | Study design | Factors that may decrease certainty of evidence | | | | | Test accuracy CoE |
| --- | --- | --- | --- | --- | --- | --- | --- |
|  |  | Risk of bias | Indirectness | Inconsistency | Imprecision | Publication bias |  |
| 2 studies 136 patients | Combination of case-control and prospective -type accuracy study | serious^a^ | not serious | serious^b^ | serious^c^ | none | ⨁◯◯◯ Very low^a,b,c^ |
|  |  |  |  |  |  |  |  |
|  |  |  |  |  |  |  |  |

#### Explanations

a. 1 study of unclear/ high risk of bias

b. Wide 95% confidence intervals in LR- and LR-

c. low number of patients

Summary of findings for: Shoulder abduction relief test

**Question**: Should shoulder abduction relief test be used to diagnose cervical radiculopathy in patients with radiating arm pain?

| № of studies (№ of patients) | Study design | Factors that may decrease certainty of evidence | | | | | Test accuracy CoE |
| --- | --- | --- | --- | --- | --- | --- | --- |
|  |  | Risk of bias | Indirectness | Inconsistency | Imprecision | Publication bias |  |
| 2 studies 159 patients | Combination of case-control, prospective-type accuracy study | serious^a^ | not serious | serious^b^ | serious^c^ | none | ⨁◯◯◯ Very low^a,b,c^ |
|  |  |  |  |  |  |  |  |
|  |  |  |  |  |  |  |  |

#### Explanations

a. one study with high risk of bias

b. wide 95% confidence intervals

c. low number of included patients

Summary of findings for: Arm Squeeze test

| № of studies (№ of patients) | Study design | Factors that may decrease certainty of evidence | | | | | Test accuracy CoE |
| --- | --- | --- | --- | --- | --- | --- | --- |
|  |  | Risk of bias | Indirectness | Inconsistency | Imprecision | Publication bias |  |
| 1 studies 1567 patients | case-control, type accuracy study | serious^a^ | not serious | not serious | very serious^b^ | none | ⨁◯◯◯ Very low^a,b^ |
|  |  |  |  |  |  |  |  |
|  |  |  |  |  |  |  |  |

#### Explanations

a. Study with high risk of bias

b. Single study both suggesting and assessing a new diagnostic test

Summary of findings for: Axial traction test

| № of studies (№ of patients) | Study design | Factors that may decrease certainty of evidence | | | | | Test accuracy CoE |
| --- | --- | --- | --- | --- | --- | --- | --- |
|  |  | Risk of bias | Indirectness | Inconsistency | Imprecision | Publication bias |  |
| 1 studies 24 patients | Prospective type accuracy study | serious^a^ | not serious | serious^b^ | very serious^c,d^ | none | ⨁◯◯◯ Very low^a,b,c,d^ |
|  |  |  |  |  |  |  |  |
|  |  |  |  |  |  |  |  |

#### Explanations

a. Study of high risk of bias

b. wide 95% confidence interval

c. low number of participants

d. single study

Summary of findings for: Neck Tornado test

| № of studies (№ of patients) | Study design | Factors that may decrease certainty of evidence | | | | | Test accuracy CoE |
| --- | --- | --- | --- | --- | --- | --- | --- |
|  |  | Risk of bias | Indirectness | Inconsistency | Imprecision | Publication bias |  |
| 1 studies 135 patients | Retrospective accuracy study type | serious^a^ | not serious | not serious | very serious^b,c^ | none | ⨁◯◯◯ Very low^a,b,c^ |
|  |  |  |  |  |  |  |  |
|  |  |  |  |  |  |  |  |

#### Explanations

a. study of high risk of bias

b. Single study both suggesting and assessing a new diagnostic test

c. low number of included patients
